# New Insights into 1-Aminocyclopropane-1-Carboxylate (ACC) Deaminase Phylogeny, Evolution and Ecological Significance

**DOI:** 10.1371/journal.pone.0099168

**Published:** 2014-06-06

**Authors:** Francisco X. Nascimento, Márcio J. Rossi, Cláudio R. F. S. Soares, Brendan J. McConkey, Bernard R. Glick

**Affiliations:** 1 Universidade Federal de Santa Catarina, Departamento de Microbiologia, Laboratório de Microbiologia do Solo, Florianópolis SC, Brazil; 2 University of Waterloo, Department of Biology, Waterloo, Ontario, Canada; Radboud University Medical Centre, NCMLS, Netherlands

## Abstract

The main objective of this work is the study of the phylogeny, evolution and ecological importance of the enzyme 1-aminocyclopropane-1-carboxylate (ACC) deaminase, the activity of which represents one of the most important and studied mechanisms used by plant growth–promoting microorganisms. The ACC deaminase gene and its regulatory elements presence in completely sequenced organisms was verified by multiple searches in diverse databases, and based on the data obtained a comprehensive analysis was conducted. Strain habitat, origin and ACC deaminase activity were taken into account when analyzing the results. In order to unveil ACC deaminase origin, evolution and relationships with other closely related pyridoxal phosphate (PLP) dependent enzymes a phylogenetic analysis was also performed. The data obtained show that ACC deaminase is mostly prevalent in some Bacteria, Fungi and members of Stramenopiles. Contrary to previous reports, we show that ACC deaminase genes are predominantly vertically inherited in various bacterial and fungal classes. Still, results suggest a considerable degree of horizontal gene transfer events, including interkingdom transfer events. A model for ACC deaminase origin and evolution is also proposed. This study also confirms the previous reports suggesting that the *Lrp-like regulatory protein AcdR* is a common mechanism regulating ACC deaminase expression in Proteobacteria, however, we also show that other regulatory mechanisms may be present in some Proteobacteria and other bacterial phyla. In this study we provide a more complete view of the role for ACC deaminase than was previously available. The results show that ACC deaminase may not only be related to plant growth promotion abilities, but may also play multiple roles in microorganism's developmental processes. Hence, exploring the origin and functioning of this enzyme may be the key in a variety of important agricultural and biotechnological applications.

## Introduction

One of the key bacterial traits in facilitating plant growth is the production of the enzyme 1-aminocyclopropane-1-carboxylate (ACC) deaminase (EC 3.5.99.7). This enzyme is responsible for the cleavage of the ethylene precursor, ACC, into ammonia and α-ketobutyrate [1]. By decreasing ACC levels in plants, ACC deaminase-producing organisms decrease plant ethylene levels [2], [3], which when present in high concentrations can lead to a reduced plant growth and ultimately, plant death [4].

ACC deaminase was initially identified in the yeast *Hansenula saturnus* (now re-classified as *Cyberlindnera saturnus*) and the bacterium *Pseudomonas* sp. ACP [1]. Since then, many groups have reported the isolation and sometimes the manipulation of *acdS* genes (i.e. the structural gene encoding ACC deaminase) from a wide range of different organisms, mostly bacteria and fungi [5]. Moreover, several studies have addressed the detailed biochemistry of ACC deaminase and the atypical and important reaction mechanism of ACC breakdown [6]. Data obtained in these studies show that ACC deaminase is a multimeric enzyme (homodimer or homotrimer) with a subunit molecular mass of approximately 35–42 kDa and it uses one molecule of pyridoxal phosphate (PLP) per subunit. Based on its protein fold, ACC deaminase has been classified as belonging to the tryptophan synthase beta superfamily (fold type II) of PLP binding proteins [6]. In this family are also included the ACC deaminase homolog from *Pyrococcus horikoshii* [7] and the D-cysteine desulfhydrase from *E.coli* and *Salmonella typhymurium* [8], [9].

ACC deaminase is central to the functional interactions of various plant associated bacteria and fungi. The root colonizing bacteria *Pseudomonas putida* GR12-2 and *Pseudomonas* sp. UW4 no longer promote canola root elongation after its *acdS* gene is knocked out [10], [11]. The symbiotic efficiency of the root nodule forming bacteria, *Rhizobium leguminosarum* bv. *viciae* and *Mesorhizobium loti* MAFF303099, is decreased upon *acdS* gene deletion [12], [13]. The endophytic plant growth-promoting bacteria *Burkholderia phytophirmans* PsJN, *Pseudomonas fluorescens* YsS6 and *Pseudomonas migulae* 8R6 are less effective when their *acdS* gene is deleted [14], [15]. Similarly, when ACC deaminase expression is impaired in the fungus *Trichoderma asperellum* T203, the plant growth promotion abilities of this organism are also decreased [16], [17].

Bacteria and fungi that express ACC deaminase can lower the impact of a range of different stresses that affect plant growth and development [3], [17]. Using ACC deaminase-producing bacteria in association with plants subjected to different kinds of biotic and abiotic stresses resulted in enhanced plant tolerance [18]–[25]. The use of ACC deaminase-producing bacteria in association with plants for purposes of soil decontamination is also documented [26]–[28]. Increased phytoremediation potential and resistance to biotic and abiotic stresses are observed in transgenic plants expressing a bacterial ACC deaminase [29]–[32]. The expression of an exogenous ACC deaminase gene increases the symbiotic performance of many rhizobial strains [33]–[36].

Studies regarding the mechanisms regulating ACC deaminase expression have been reported for some Proteobacteria. Binding sites for CRP (cAMP receptor protein), FNR (fumarate-nitrate reduction regulatory protein) and LRP (leucine responsive regulatory protein) were present in the promoter region of the *Pseudomonas* sp. UW4 *acdS* gene and were shown to function in regulating *acdS* expression [37]–[39]. In addition, an LRP-like protein-coding region has been found in the immediate upstream region of many *acdS* genes. This gene was termed *acdR* (ACC deaminase regulatory protein), based on the evidence that it is necessary for optimum ACC deaminase expression in the presence of ACC. The *acdR* gene has also been demonstrated to participate in the regulation of ACC deaminase expression in *Rhizobium leguminosarum* bv. *viciae* 128C53K and *Azospirillum lipoferum* 4B [12], [40]. Most other Proteobacteria that have been examined for ACC deaminase activity or *acdS* gene presence, possess an *acdR* gene in the vicinity of *acdS*, suggesting that this regulatory mechanism is widespread in *acdS*+ Proteobacteria [40].

Despite the fact that many biochemical and biological features of ACC deaminase are now understood, not much is known about the origin and phylogeny of the *acdS* gene and its regulatory elements. Based upon a phylogenic analysis of a limited number of *acdS* genes partially characterized and their comparison to the phylogeny of 16S rRNA genes from the same bacteria, Hontzeas et al. [41] proposed that some ACC deaminase genes have been transmitted through horizontal gene transfer (HGT). Using the same criteria, Blaha et al. [42] suggested that ACC deaminase genes in Proteobacteria were extensively subjected to HGT. In addition, Nascimento et al. [43] suggested that in many *Mesorhizobium* spp. the *acdS* gene is transferred between strains through symbiotic island exchange.

The phylogeny in Proteobacteria of *acdR* has also been investigated. Prigent-Combaret et al., [40] suggested that *acdR*, like *acdS*, may have evolved through HGT. This conclusion notwithstanding, these authors suggest that the evolution of *acdS* and *acdR* genes might not be coupled.

While phylogenetic studies of *acdS* and *acdR* genes have been focused primarily on Proteobacteria, other studies have demonstrated the presence of ACC deaminase activity in Actinobacteria [41], [44]–[48], Firmicutes [44], [48]–[51] and Bacteroidetes [52]–[55]. Furthermore, the presence of a putative functional ACC deaminase in *Phytophthora sojae* [56] further emphasizes the notion that the current view of *acdS* phylogeny and evolution is somewhat incomplete. To address this, here we have undertaken a comprehensive study of the phylogeny of *acdS* and *acdR* and the results are discussed in terms of evolutionary and ecological implications of ACC deaminase production by diverse microorganisms.

## Methods

### Obtaining the sequences

To obtain bacterial ACC deaminase (AcdS) and ACC deaminase regulatory protein (AcdR) sequences, BLAST searches were performed in the NCBI databases (www.ncbi.nlm.nih.gov/) using *Pseudomonas sp*. UW4 *acdS* and *acdR* gene, as well as AcdS and AcdR protein sequences as the queries. For fungal ACC deaminase sequence retrieval, BLAST searches were performed in the NCBI database using the *Penicillium citrinum* AcdS protein sequence as the query. Default BLAST parameters were used when obtaining the sequences.

An NCBI genomic BLAST search (www.ncbi.nlm.nih.gov/sutils/genom_table.cgi) was also performed using *Pseudomonas* sp. UW4 *acdS* and AcdS sequences in order to evaluate the presence of ACC deaminase in other completely sequenced organisms. An additional BLAST search was performed in the nematode genomic database (www.nematodes.org) using *Pseudomonas* sp. UW4 or *Penicillium citrinum acdS* gene as query.

Moreover, all putative AcdS sequences were analyzed for key protein residues known to be important for ACC deaminase activity, namely Lys51, Ser78, Tyr295, Glu296 and Leu322 [7], [57], [58] using *Pseudomonas* sp. UW4 as a reference. The AcdS sequences were aligned using MUSCLE [59] and the presence of key amino acid positions were verified. Sequences presenting different amino acids in the above mentioned positions were discarded, as they are likely to represent related PLP dependent enzymes, such as D-cysteine desulfhydrase [58].

Sequence identities and similarities were analyzed using SIAS (http://imed.med.ucm.es/Tools/sias.html) with default parameters.

When available, the genomic regions containing the *acdS* gene were analyzed in order to identify any patterns present in the *acdS* gene neighborhood.

Strain information and 16S rRNA gene sequences were obtained via NCBI (http://www.ncbi.nlm.nih.gov), Goldcard (http://www.genomesonline.org/cgi-bin/GOLD/index.cgi) and SILVA (http://www.arb-silva.de), where available.

The accession numbers for sequences used in this study as well as strains descriptions are presented in Tables S1 (Actinobacteria, Deinococcus-Thermus and Firmicutes), S2 (α-Proteobacteria), S3 (β-Proteobacteria), S4 (γ-Proteobacteria), and S5 (Eukaryotes).

### ACC deaminase protein sequence analysis and comparison to closely related enzymes

Protein sequence analysis was conducted on AcdS proteins found in completely sequenced representative bacteria. The functional AcdS protein sequences of the Proteobacteria *Agrobacterium tumefaciens* D3 [60], *Azospirillum lipoferum* 4B [40], *Bradyrhizobium japonicum* USDA110 [61], *Mesorhizobium loti* MAFF303099 [13], *Phyllobacterium brassicacearum* STM196 [62], *Rhizobium leguminosarum* 128C53K [12], *Sinorhizobium meliloti* SM11 [63], *Burkholderia phytofirmans* PsJN [14], *Burkholderia graminis* C4D1M [64], *Ralstonia solanacearum* GMI1000 [42], *Variovorax paradoxus* 5C2 [22], *Pseudomonas* sp. UW4 [38], *Pseudomonas* sp. ACP [1] and the Fungi, *Cyberlidnera saturnus* [1], *Penicillium citrinum* [65], *Trichoderma asperellum* T203 [16], together with the AcdS from *Herbaspirillum frinsigense* GSF30 [66] and the putative AcdS sequences from *Agreia* sp. PHSC20C1, *Rhodococcus* sp. R04 (Actinobacteria), *Meiothermus ruber* DSM1279 (Deinococcus-Thermus) were used. Sequences were aligned using MUSCLE and the presence of conserved and variable sites was analyzed.

Sequence comparisons were also performed with closely related enzymes. Therefore, D-cysteine desulfhydrase sequences from *E. coli* [8], as well as the ACC deaminase homologs from *Pyrococcus horikoshi* [7] and *Solanum lycopersicum* [58] were used and compared to the various ACC deaminase proteins.

### Phylogenetic analysis

The sequences were aligned using MUSCLE and phylograms were constructed in Seaview v.4.2.12 [67] using PhyML [68].

In order to obtain the best substitution model for the construction of the phylogenetic trees, the resulting alignments were analyzed with jModeltest2 [69] and ProtTest [70]. The substitution models were chosen based on minimum BIC (Bayesian Information Criteria) values.

The *acdS*, *acdR* and 16S rRNA gene evolutionary history was inferred by using the Maximum Likelihood method based on the GTR model with a discrete Gamma distribution (4 Gamma categories). The AcdS and AcdR phylograms were constructed using the Maximum likelihood method based on the WAG model with a discrete Gamma distribution (4 Gamma categories). Branch support was evaluated using both aLRT (SH like) [71] and bootstrap analysis (100 replicates). Only bootstrap values above 0.75 (75%) are included in the phylograms. The resulting phylogenetic trees were plotted using FigTree v.1.4.1 (http://tree.bio.ed.ac.uk/software/figtree).

Estimates of evolutionary divergence between *acdS* sequences or 16S rRNA sequences in groups of bacterial strains were computed using MEGA software 6.06 [72]. The number of base substitutions per site from between sequences was calculated and analyses were conducted using the Maximum Composite Likelihood model with 1000 bootstrap replications. The analysis involved 3 nucleotide sequences per group of bacterial species, previously aligned using MUSCLE. Codon positions included were 1st+2nd+3rd+Noncoding. All positions containing gaps and missing data were removed.

## Results and Discussion

### ACC deaminase prevalence in completely sequenced organisms

After performing multiple searches in the NCBI database (http://www.ncbi.nlm.nih.gov/sutils/genom_table.cgi) using *Pseudomonas* sp. UW4 *acdS* gene as query, it was observed that the *acdS* gene is not commonly seen in most sequenced organisms. The *acdS* gene is mainly found in Actinobacteria, members from the Deinococcus-Thermus phylum (*Meiothermus*), three classes from Proteobacteria (α, β and γ), in various Fungi classes belonging to Ascomycota and Basidiomycota, and in Stramenopiles members.

These results are in agreement with previous reports, which have demonstrated ACC deaminase activity in many Actinobacteria, α, β and γ-Proteobacteria. Remarkably, putative *acdS* genes were found in *Meiothermus*, yet, there is no record of ACC deaminase activity in these thermophile strains. Putative *acdS* genes were also found many in members of Stramenopiles, mostly in *Phytophthora*. By computational analysis, Singh and Kashyap, [56] suggest that the *acdS* gene found in *Phytophthora sojae* encodes a functional ACC deaminase.

Interestingly, despite the known ACC deaminase activity display by bacteria belonging to the Bacteroidetes/Chlorobi or Firmicutes, it was not possible to identify *acdS* genes in the completely sequenced bacteria belonging to these phyla. In 478 completely sequenced bacteria (accessed in July, 2013) belonging to the Bacteroidetes/Chlorobi, including many *Flavobacterium* and *Chryseobacterium* species, the *acdS* gene is not found. Although candidate *acdS* genes are identified via BLAST, the active sites contain residues more consistent with D-cysteine desulfhydrase or a related PLP dependent enzyme [58], such as YP_001296100 in which threonines replace residues corresponding to active site residues E296 and L322. ACC deaminase activity has been previously reported to be present in *Flavobacterium* and *Chryseobacterium* species, although at very low levels [52], [55], which may represent non-specific activity of D-cysteine desulfhydrase-like enzymes. Similarly, although ACC deaminase activity has been described in many *Bacillus* and *Paenibacillus* strains [48]–[51], it was not possible to identify the *acdS* gene in 271 completely sequenced strains belonging to the Bacilli class (Firmicutes phylum), including many soil and plant associated *Bacillus* and *Paenibacillus* species.

It is possible that in these and many other bacterial strains the presence of an *acdS* gene may be related to a strain's specific feature in which *acdS* acquisition happened by HGT by result of a co-existence with other ACC deaminase-producing bacteria in environments where ACC deaminase production provides the bacteria with some important advantages. Other possible explanations for this inconsistency may relate to the fact that genome sequencing is biased and the sequenced strains may not be representative of bacteria that interact extensively with plants.

### Analysis of ACC deaminase (putative and functional) protein sequences

In the first instance, every sequence used in this study (Table S1–S5) contains the previously described AcdS conserved regions that have been found to be necessary for ACC deaminase activity. Moreover, all bacterial AcdS sequences shared high sequence identity (60 to 100%) to AcdS from *Pseudomonas* sp. UW4.

When comparing the putative AcdS sequences from Fungi with the functional ACC deaminase from *Penicillium citrinum*, sequence identities ranged between ∼70 and 99% for the majority of fungal AcdS sequences. Exceptionally, some AcdS sequences from yeasts and some other fungi share only ∼52–55% identity to *Penicillium citrinum* AcdS. Also, the Stramenopiles members share approximately 60% identity to the *Penicillium citrinum* AcdS. Interestingly, the AcdS sequences from yeasts, some other Fungi and Stramenopiles share higher identity to *Pseudomonas* sp. UW4 AcdS sequence (∼70 to 85%), consistent with a relationship with Proteobacteria and the possibility of past horizontal gene transfers. A more detailed description of this issue is presented below.

Protein sequence analysis suggests that the putative *acdS* genes found in *Rhodococcus* sp. R04, *Agreia* sp., PHSC20C1 and *Meiothermus ruber* DSM1279, encode a true ACC deaminase. By sequence comparison, it was observed that the putative AcdS contain all the conserved features present in all known functional ACC deaminases and not present in the related enzymes (Figure S1). For instance, the putative AcdS sequences contain the important residues E295 and L322 known to be required for ACC deaminase activity [58] and not present in other related enzymes. These results are also supported by the fact that these Actinobacteria and *Meiothermus* AcdS protein sequences share high identity (70 to 82%) to the functional ACC deaminase from *Rhodococcus* sp. 4N-4 (partially characterized) [41]. In addition, these sequences show similar sequence identities to other β and γ-Proteobacteria AcdS sequences (∼70%).

### ACC deaminase phylogeny: Horizontal gene transfer or vertical transmission?

**Figure 1:**
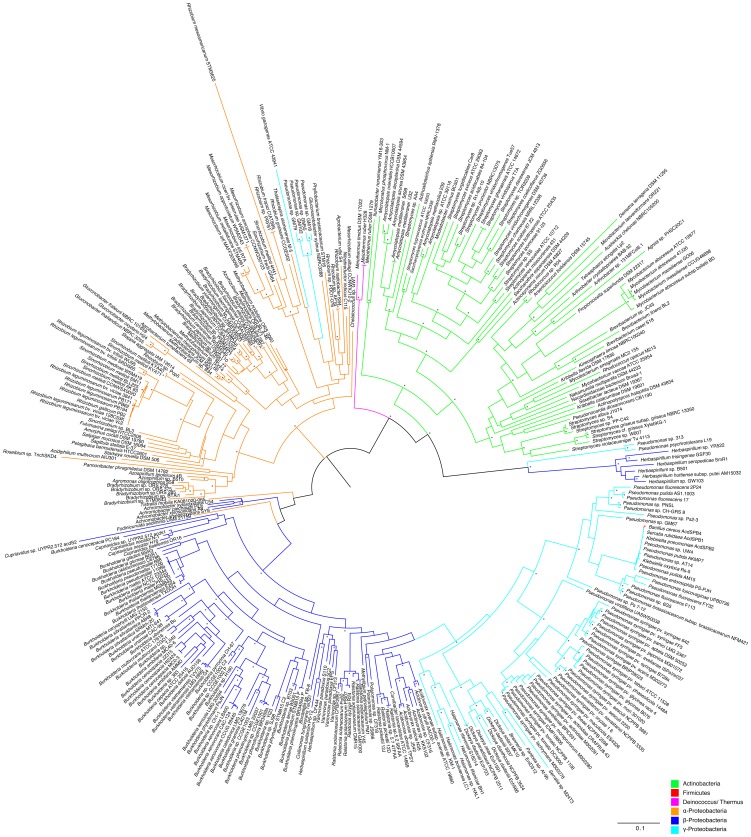
Phylogram based on the *acdS* gene. The evolutionary history was inferred by using the Maximum Likelihood method based on the GTR model. A discrete Gamma distribution was used to model evolutionary rate differences among sites (4 categories). Branch support was evaluated using both aLRT (SH like) and bootstrap analysis (100 replicates). Bootstrap values above 0.75 (75%) are displayed in the phylograms shown next to the branches as *. The analysis involved 335 nucleotide sequences and 931 patterns were found (out of a total of 1155 sites).

**Figure 2:**
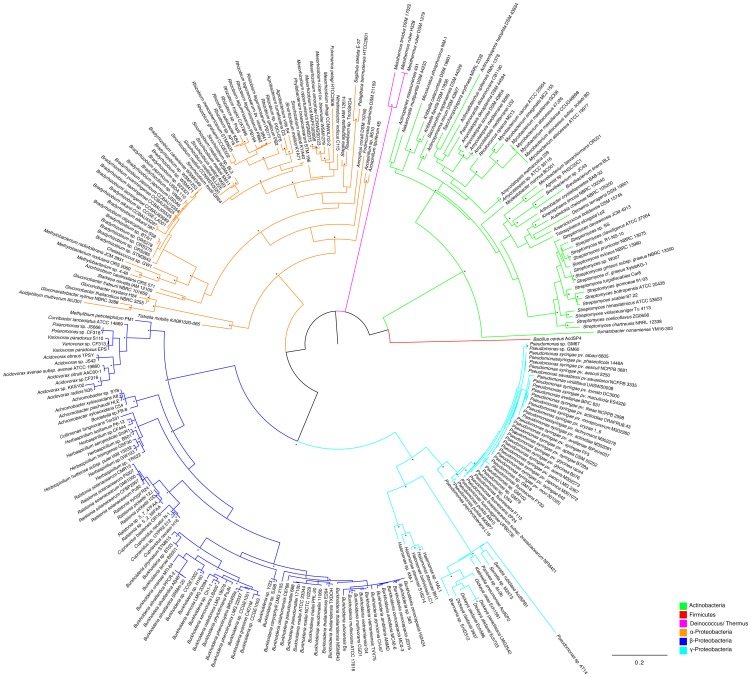
Phylogram based on 16S rDNA sequences. The evolutionary history was inferred by using the Maximum Likelihood method based on the GTR model. A discrete Gamma distribution was used to model evolutionary rate differences among sites (4 categories). Branch support was evaluated using both aLRT (SH like) and bootstrap analysis (100 replicates). Bootstrap values above 0.75 (75%) are displayed in the phylograms shown next to the branches as *. The analysis involved 272 nucleotide sequences and 768 patterns were found (out of a total of 1334 sites).

The comparison between the *acdS* phylogenetic tree (Fig. 1) and the 16S rRNA based phylogeny (Fig. 2), suggests that ACC deaminase has evolved mainly through vertical transmission with occasional horizontal gene transfer. In the *acdS* phylogram (Fig. 1), it is observed that closely related strains typically have similar *acdS* gene sequences. Furthermore, many strains with different origins and isolated from different habitats (Tables S1–S5), but belonging to the same species tend to have similar *acdS* genes.

The presence of the *acdS* gene in an organism like *Meiothermus ruber* is also consistent with the vertical transmission of this gene. It is unlikely that this bacterial thermophile (optimum growth at 60°C) isolated from a hot spring has acquired an *acdS* gene through HGT. This is strongly supported by the *acdS* gene phylogram (Fig. 1) showing a well bootstrap-supported and unique cluster grouping all *Meiothermus acdS* sequences distantly from all other *acdS* genes obtained from different bacterial phyla.

The presence of an *acdS* gene in the chromosome of the psychrophile marine actinobacterium, *Agreia* sp. PHSC20C1, (isolated in the Antarctic) and other soil Actinobacteria, is also consistent with the vertical transmission and ancient origin of the *acdS* gene. In *Azorhizobium* and *Bradyrhizobium* strains, the *acdS* gene is located far away from the “plastic” chromosomal symbiotic island containing the symbiotic genes. If these strains had acquired the *acdS* gene by HGT it might be expected that it would be present in a region that is more prone to such transfers, such as a symbiotic island or a plasmid.

Blaha et al. [42] and Glick et al. [3] have suggested that that the presence of *acdS* on plasmids may facilitate the lateral transfer of this gene. On the other hand, the presence of the *acdS* gene on a plasmid can also account for a different sequence divergence rate. Mobile elements and smaller replicons show higher evolutionary rates when compared to primary chromosomes [73], [74]. By being present on smaller replicons, *acdS* genes may be subject to different evolutionary rates compared to genes present in primary chromosomes. This may help to explain the *acdS* gene phylogeny of *Burkholderia* and *Cupriavidus*. Instead of clustering together with the other β-Proteobacteria, strains belonging to the *Burkholderia* and *Cupriavidus* genus form a separate cluster (Fig. 1). From the available data, most *Burkholderia* and *Cupriavidus* strains have the *acdS* gene present in a second smaller chromosome. Other β-Proteobacteria possesses an *acdS* gene in the primary chromosome or in plasmids (Table S3). This phenomenon is also observed in *Agrobacterium* and *Rhizobium* strains, *A. vitis* S4 and *R. radiobacter* K84 which have the *acdS* gene located in a second chromosome, and therefore, cluster distantly from their *Agrobacterium tumefaciens* D3 (*acdS* in plasmid) and *Rhizobium* (*acdS* in plasmid) relatives. Thus, there seems to be a connection between *acdS* phylogenetic distribution, evolution and *acdS* location in the replicon.

Environmental cues can also lead to different gene mutation rates [75]. Gene loss, acquisition, mutational rates and genome rearrangements may play a crucial role in bacterial adaptation and survival [76], [77]. This is particularly important in organisms living in adverse environments like many of the organisms described here (Tables S1–S5). It is possible that bacteria adapted to different environments may present different *acdS* divergence rates, thus being responsible for some of the variance in *acdS* genes in bacteria from the same species. When calculating the 16S rRNA and *acdS* gene evolutionary distance estimates in specific bacterial species groups it was found that the ratio between 16S rRNA and *acdS* sequence divergence is not always identical between strains and groups (file S1). For instance, three *Burkholderia mallei* strains isolated from three different countries show identical 16S rRNA (1200 bp) (d = 0) and identical *acdS* gene (1019 bp) (d = 0) sequences. In three *Burkholderia silvatlantica* strains obtained from Brazil, this is not observed; all strains present identical 16S rRNA sequences (1200 bp) (d = 0) but show intraspecific differences in the *acdS* gene sequences (1019 bp) (d = 0.0059±0.0020), sometimes accounting to up to 5 different nucleotides. Interestingly, all three *B. mallei* were obtained from human and animal blood and are known pathogens, while the three *B. silvatlantica* strains were obtained from the rhizosphere of different plants where they act like plant growth-promoting bacteria [64].

Several authors suggested HGT for *acdS* genes based on results showing a specific relative position of some *Pseudomonas* (γ-Proteobacteria) strains in the *acdS* phylogenetic tree [41], [42], [6]. Instead of forming a separate cluster, some *Pseudomonas* strains clustered together with β-Proteobacteria. In this work, we obtained somewhat similar results. Although members of γ-Proteobacteria group very close to β-Proteobacteria, they form a unique cluster and are not scattered through the phylogenetic tree as observed in previous studies. A very close evolutionary relationship between these two classes has been reported [78]–[81]. In fact, some bacterial strains that belonged to the *Pseudomonas* genus (γ-Proteobacteria) have been reassigned to the *Burkholderia* genus (β-Proteobacteria) [82], [83].

**Figure 3:**
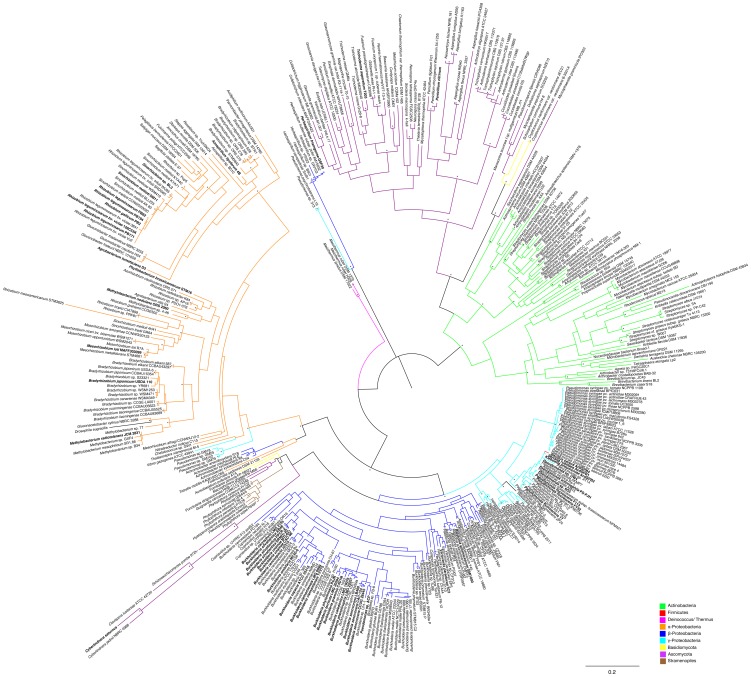
Phylogram based on AcdS protein. The evolutionary history was inferred by using the Maximum Likelihood method based on the WAG model. A discrete Gamma distribution was used to model evolutionary rate differences among sites (4 categories). Branch support was evaluated using both aLRT (SH like) and bootstrap analysis (100 replicates). Bootstrap values above 0.75 (75%) are displayed in the phylograms shown next to the branches as *. The analysis involved 431 amino acid sequences and 386 patterns were found (out of a total of 421 sites). Functional ACC deaminases are shown in bold.

Interestingly, the *Pseudomonas* sp. ACP AcdS sequence shares higher identity (96.7%) with *Burkholderia xenovorans* LB400 functional ACC deaminase than with *Pseudomonas* sp. UW4 AcdS (85.3%). This is also observed in the AcdS phylogram, where *Pseudomonas* sp. ACP groups closer to *Burkholderia xenovorans* LB400 (Fig. 3). While, Honma and Shimomura [1] tentatively identified *Pseudomonas* sp. ACP bacterium by phenotypic methods, it is conceivable that *Pseudomonas* sp. ACP is in fact a *Burkholderia* strain [64]. If this is in fact the case, then previous studies regarding the phylogeny of *acdS* may also have been influenced by the confusing relationship between *Pseudomonas* and *Burkholderia*. Furthermore, due to the recent divergent evolution and close relationship between γ and β-Proteobacteria, it is very difficult to prove *acdS* HGT in these classes.

While less prevalent than previously thought, HGT likely does occur and accounts for a portion of *acdS* gene evolution. For example, it has been shown that some *Mesorhizobium* strains may acquire a specific *acdS* gene by the means of symbiotic island transfer [43]. Nandasena et al., [84] demonstrated that *Mesorhizobium opportunistum* WSM 2073 acquired a specific symbiotic island when it came in contact with non-endemic populations of *M. ciceri* bv. *biserrulae*, thus, gaining the ability to nodulate *Biserrula pelecinus*. The *acdS* gene was present within that symbiotic island and was therefore transferred between these strains. Moreover, the *acdS* gene sequences from those two strains share 100% identity, strongly supporting the idea of a recent transfer event.

Curiously, there are some cases where *acdS* horizontal transfers seem to have occurred between strains with a more distant evolutionary relationship. This is the case of *Pseudomonas* isolates GM 18, GM 55, GM 79 and GM 102, which are found to possess *acdS* genes like those of α-Proteobacteria (Fig. 1). Despite belonging to the γ-Proteobacteria, *Vibrio gazogenes* ATCC43941 has an *acdS* gene resembling those of α-Proteobacteria (Fig. 1). Chen et al., (2013) showed that *Bacillus cereus* AcdSPB4 isolated from the casing soil of *Agaricus bisporus* possesses an *acdS* gene highly similar to those of *Pseudomonas* (Fig. 1) thus, strengthening the idea of *acdS* horizontal transfer between distantly related strains.

Interestingly, *Herbaspirillum seropedicae* SmR1, *H. frisingense* GFS30, *H. huttiense* subsp. *putei* AM15032, *H.* sp. B501, *H.* sp. GW103, *H.* sp. YR522, *Pseudomonas psychrotolerans* L19 and *Pseudomonas* sp. 313 strains possess *acdS* genes that are not similar to those found in other bacteria from the same Class or even to other *Herbaspirillum* and *Pseudomonas* strains. Instead they form a unique group in the phylogenetic tree (Fig. 1, 3). Furthermore, these strains also possess *acdR* genes that are frequently found in AcdS+ Proteobacteria. In this scenario it is possible that these strains have horizontally acquired *acdS* and *acdR* genes from a different class of bacteria yet to be determined. One may also assume that the putative *acdS* genes in these strains encode a different type of deaminase, however, ACC deaminase activity has been detected in *Herbaspirillum frisingense* GFS30 [66]. Moreover, the putative AcdS from *H. frisingense* GFS30 shows the conserved regions known to be important in functional ACC deaminases (i.e E295, L322) (Fig. S1). Curiously, *Herbaspirillum frisingense* GFS30 and *Herbaspirillum* sp. YR522 are both endophytes isolated from *Miscanthus* and *Populus deltoides,* respectively.

Similar to what is observed in Bacteria, the AcdS phylogeny in Fungi indicates that closely related strains possess a similar ACC deaminase (Fig. 3). This is consistent with the notion that *acdS* genes are vertically transmitted in Fungi. However, some fungal strains like *Penicillium marneffei* and *Talaromyces stipitatus* (Ascomycota/Eurotiomycetes) are likely to have acquired the *acdS* from other Fungi belonging to the Sordariamycetes class, suggesting that like in some bacteria, fungal *acdS* genes may also be acquired by HGT.

In addition, the yeasts *Cyberlindnera saturnus*, *Cyberlindnera jadinii* NBRC 0988, *Clavispora lusitaniae* ATCC 42720 (Ascomycota/Saccharomycetes) and *Schizosaccharomyces pombe* 972h- (Ascomycota/Schizosaccharomycetes) seem to have acquired an ACC deaminase gene separately from most Ascomycota and presumably from Proteobacteria. ACC deaminase genes like those of Proteobacteria have also been detected in Fungi belonging to different classes such as *Punctularia strigosozonata* HHB-11173 (Basidiomycota/Agaricomycetes), *Fomitopsis pinicola* FP-58527 (Basidiomycota/Agaricomycetes), *Aureobasidium pullulans* AY4 (Ascomycota/Dothideomycetes), *Macrophomina phaseolina* MS6 (Ascomycota/Dothideomycetes) and *Guignardia citricarpa* CGMCC3.14348 (Ascomycota/Dothideomycetes).

The Stramenopiles, Phytophthora infestans T30-4, P. ramorum Pr102, P. sojae P6497, P. lateralis, P. kernoviae, P. parasitica, Pseudoperonospora cubensis and Hyaloperonospora arabidopsidis Emoy2 also have ACC deaminase genes most similar to those of Proteobacteria (Fig. 3).

Searches of diverse genomic databases also have revealed the presence of putative *acdS* genes in other eukaryotic organisms like the nematode *Howardula aoronymphium* and the fly *Drosophila eugracilis*. Furthermore, these genes show high similarity to *acdS* from Proteobacteria (Fig. 3). Some *acdS* genes are found in bacteria known to be associated with Eukaryotic organisms, for example, *Serratia* sp. M24T3, isolated from the nematode *Bursaphelenchus xylophilus,* and *Pantoea* sp. At-9b, the leaf cutter ant symbiont (Table S4). While it is possible that *Howardula aoronymphium* and *Drosophila eugracilis* may have acquired *acdS* genes from associated bacteria, it is most likely that the presence of *acdS* in these organisms results from contamination of genomic DNA.

### AcdR phylogeny: Have AcdR and AcdS undergone a coupled evolution?

In the study conducted by Prigent-Combaret et al. [40], 45 of 48 studied Proteobacteria were found to possess an LRP homolog (*acdR*) near the *acdS* gene. Here, we report the presence of *acdR* in, at least, 166 of 261 Proteobacteria possessing an *acdS* gene. Still, it was not possible to obtain the *acdR* sequence in many (78) organisms and others only have their *acdS* gene described. The *acdR* gene was not found at least in 17 *acdS*+ completely sequenced strains (6 *Mesorhizobium* strains, 2 *Rhizobium* strains, *Fulvimarina pelagi*, 3 root nodule *Burkholderia* strains containing the 2^nd^ *acdS* copy in a plasmid, *Halomonas titanicae* BH1 and 4 *Pseudomonas* strains).

**Figure 4:**
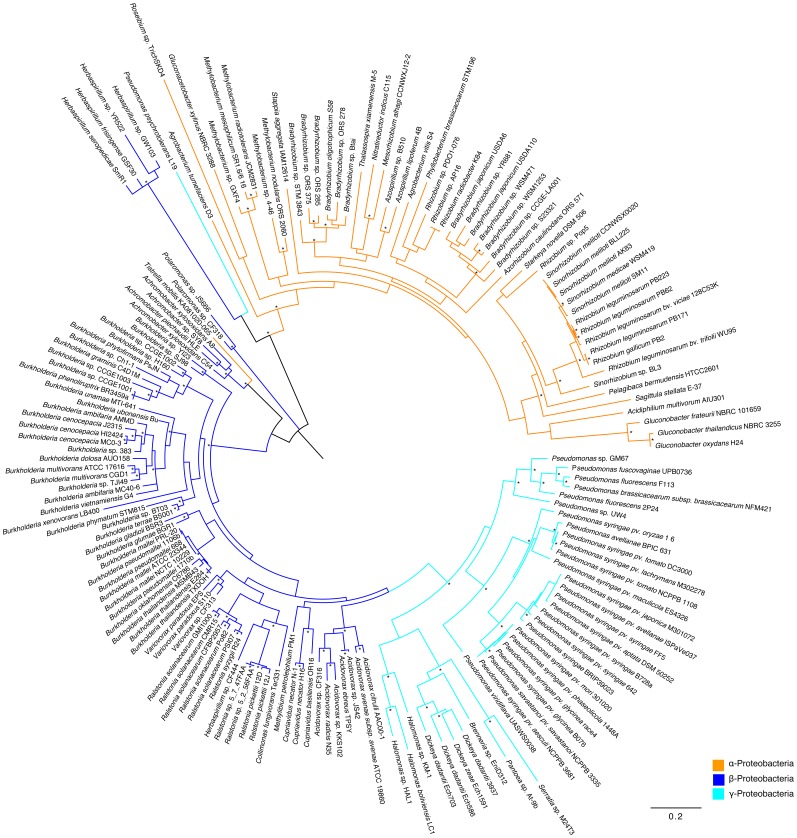
Phylogram based on *acdR* gene. The evolutionary history was inferred by using the Maximum Likelihood method based on the GTR model. A discrete Gamma distribution was used to model evolutionary rate differences among sites (4 categories). Branch support was evaluated using both aLRT (SH like) and bootstrap analysis (100 replicates). Bootstrap values above 0.75 (75%) are displayed in the phylograms shown next to the branches as *. The analysis involved 166 nucleotide sequences and 509 patterns were found (out of a total of 594 sites).

Moreover, the *acdR* gene is found in the opposite direction of the *acdS* gene in most studied Proteobacteria (data not shown). This is consistent with the previous reports of Grichko and Glick [37], Ma et al. [12] and Prigent-Combaret et al. [40]. This data suggest that *acdR* is a common mechanism regulating ACC deaminase expression in most Proteobacteria. Moreover, the phylogeny of *acdR* (Fig. 4) is related to the *acdS* gene phylogeny (Fig. 1), suggesting that these genes evolved in a similar and dependent manner. Closely related strains have similar *acdR* genes, as also observed in the *acdS* phylogram, suggesting that *acdR* is primarily vertically inherited. In the phylogram based on the *acdR* gene it is also observed a grouping according to the bacterial Class (taxonomy) and the gene location in the replicon (Example: 2nd chromosome location in *Burkholderia* and *Cupriavidus* vs. primary chromosome/plasmid location in other β and γ-Proteobacteria).

Interestingly, there are few cases where it seems that the *acdR* and *acdS* are not inherited together or may have undergone genomic rearrangements. While some strains don't have *acdR* genes in the vicinity of the *acdS* gene others don't have an *acdR* gene at all. In *Gluconacetobacter xylinus* NBRC 3288 there are various regions coding putative LRP in the upstream region of the *acdS* gene. However, they are not true *acdR* genes. A sequence sharing high homology to the *acdR* gene is found far away (aprox. 9 kb) from the *acdS* gene. This is also observed in *Burkholderia xenovorans* LB400. In this case, despite the fact that this strain has an *acdR* gene located far from the *acdS* gene, it is still able to express ACC deaminase [64]. In *Rhizobium leguminosarum* bv. *viciae* 3841 an *acdR* gene is not found. In *Mesorhizobium loti* MAFF303099 the *acdR* gene is also not present, but in this case, the *acdS* gene transcription is regulated by NifA [13].

It is possible that genome rearrangements or gene insertions in smaller replicons can account for the absence of *acdR* genes in some *acdS*+ bacterial strains. The strains *Burkholderia* sp. CCGE1002, *B. phymatum* STM815 and *B. phenoliruptrix* BR3459a (isolated from root nodules) have two copies of the *acdS* gene, one on the second chromosome and the other on a megaplasmid. The *acdS* gene copy present on the megaplasmid seems to be the result of *acdS* gene duplication and later insertion into this smaller replicon. This is consistent with the high identity between the two *acdS* copies and also the presence of transposase genes in the immediate upstream and downstream regions of the *acdS* gene. In this case, the *acdR* gene is not present and may have been lost in this process.

The exceptions notwithstanding, in the majority of cases in Proteobacteria it appears that the evolution of *acdS* and *acdR* is coupled. This result is in agreement with previous reports showing that *acdR* is necessary for optimum ACC deaminase expression [38], [39].

Despite being mostly inherited together, it is observed that these genes may have different evolutionary rates. Thus, for example, compared to the *Pseudomonas* sp. UW4 AcdR sequence, other AcdR sequences from Proteobacteria show identities ranging from 51% to 87%. This degree of variability is not observed in Proteobacteria AcdS sequences. By coding a regulatory protein it is most likely that the *acdR* gene is more prone to modifications, thus, allowing fine-tuning of *acdS* transcription and expression.

Previously, Nikolic et al. [85] stated that “the *acdR*–*acdS* gene cluster is rather rare and typically occurs in few α and β-Proteobacterial genera” based on finding the *acdR*–*acdS* gene cluster in four α, six β-Proteobacteria and in only three *Pseudomonas syringae* strains. They concluded that the operon is rather uncommon among γ-Proteobacteria. However, more detailed data presented by Prigent-Combaret et al. [40] and also in this study, supports a widespread occurrence of *acdR*–*acdS* gene cluster. The apparent lack of *acdR*-*acdS* clusters in Nikolic et al. [85] may be due to the inclusion of putative ACC deaminase sequences that were not confirmed by comparison with conserved protein domains. Thus, sequences coding for D-cysteine desulfhydrases, and possibly other deaminases and aminotransferases, were considered as ACC deaminases, leading to a confusing relationship between *acdS* and *acdR* and also the presence of *acdS* in some bacterial groups.

### Other mechanisms regulating ACC deaminase transcription

The expression of ACC deaminase by organisms that don't possess *acdR* genes indicates that the presence of this regulator is not absolutely necessary for *acdS* transcription. The presence of CRP and FNR binding sites in the immediate upstream region of the *acdS* gene in many Proteobacteria [37], [40] suggests that these elements can also account for ACC deaminase expression regulation in some Proteobacteria. It has been demonstrated directly in some instances that FNR as well as CRP regulate *acdS* transcription [37], [38], [40]. The NifA protein is also a known regulator of ACC deaminase expression in *Mesorhizobium loti* MAFF303099. In this strain ACC deaminase expression occurs only inside of formed nodules [13], [86]. In addition, a NifA binding site is found in the immediate upstream region of the *acdS* gene in this and many other *Mesorhizobium* strains, suggesting that this regulatory mechanism is widespread in this genus [43]. Interestingly, the NifA binding site (5′-TGT-N_9–11_-ACA-3′) is quite similar to the CRP binding site (5′-TGTGA-N_6_-TCACA-3′).

In many Actinobacteria and in *Meiothermus*, a gene encoding a protein from the GntR family of transcriptional regulators is found next to the *acdS* gene. We putatively termed it *acd-AR* (Actinobacteria) and *acd-MR* (*Meiothermus*). When performing BLAST searches using one Acd-AR protein sequence as query, the main hits are always related to other Acd-AR protein sequences found in *acdS*+ Actinobacteria, suggesting a close relationship between *acdS* and *acd-AR*. The same trend is observed in *Meiothermus* despite the fact Acd-AR shares low identity to Acd-MR. There are no sequences in the database that share a high degree of similarity to Acd-MR. These results are consistent with the possibility that both *acd-AR* and *acd-MR* might be involved in the regulation of ACC deaminase expression in these organisms. Curiously, when analyzing the immediate upstream region of the *acdS* gene in various Actinobacteria it is observed that some strains appear to have no promoter regions (Fig. S2-A). The same is observed in *Meiothermus*. In these strains the *acdS* gene forms an operon together with the *acd-AR* gene and *acd-MR* gene, respectively.

Interestingly, in Nocardioidaceae Broad-1 a leucine responsive protein is found in the vicinity of the *acdS* gene, however, it is quite different from the Proteobacteria AcdR protein. Also, in some Actinobacteria and Proteobacteria strains the *acdS* gene is located near a transcriptional regulator belonging to the LysR family. Moreover, in *Saccharopolyspora erythraea* NRRL 233 and *Streptomyces hygroscopicus* ATCC 53653 strains the *acdS* gene also appears to be part of an operon consisting of a gene encoding a MFS family protein and another gene encoding a M20 peptidase (Fig. S2-B). Interestingly, a LysR transcriptional regulator is also found in the vicinity of the *acdS* gene in some Proteobacteria like *Brenneria* sp. EniD312, *Burkholderia xenovorans* LB400, *Dickeya* spp. and *Pantoea* sp. At-9b (Fig. S2-C-H). The presence of peptidase M20 in the vicinity of *acdS* is also observed in some of these strains.

Further studies are necessary in order to characterize the importance of these regulators in ACC deaminase expression in different organisms.

### ACC deaminase origin

**Figure 5:**
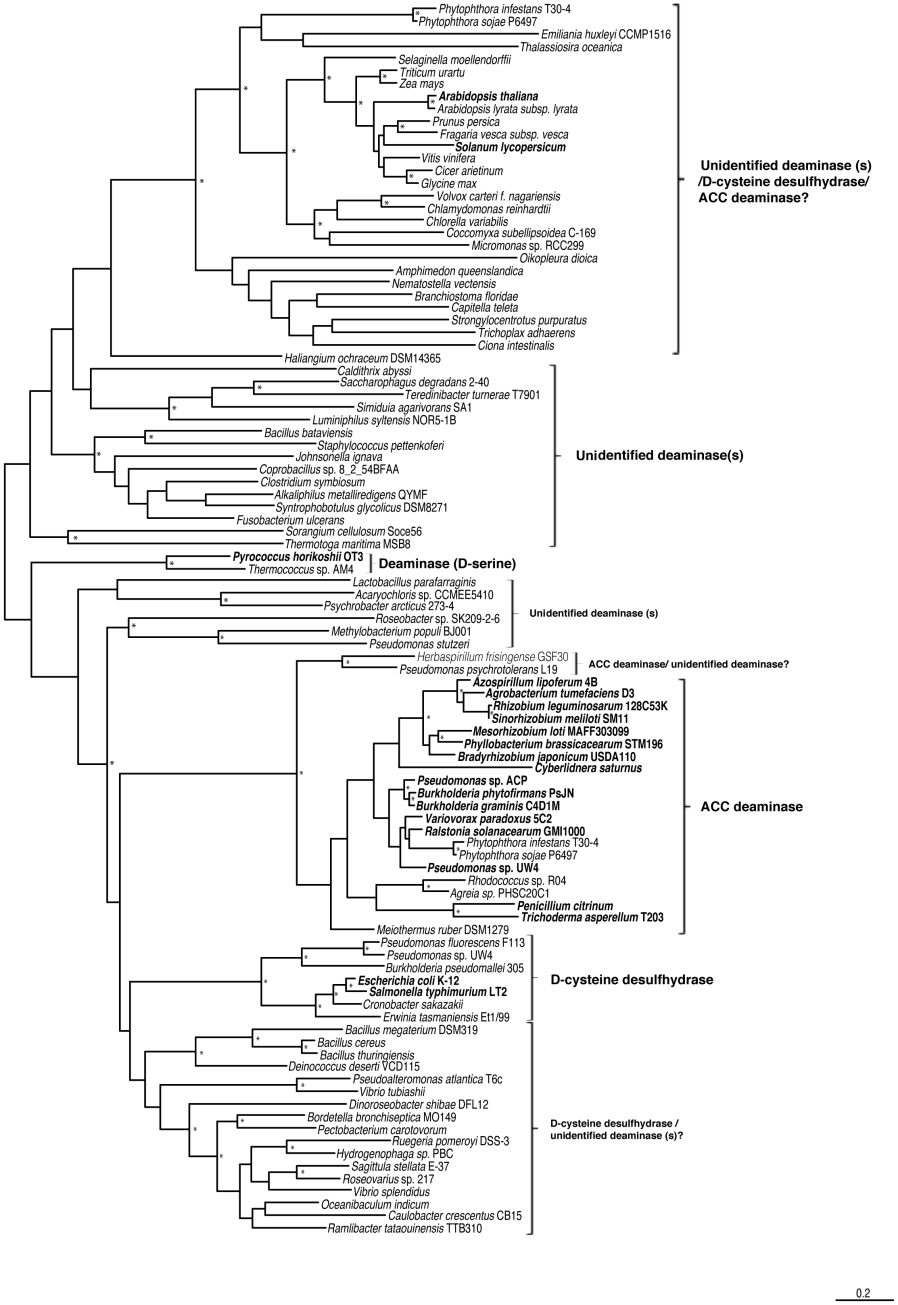
Phylogram constructed based on ACC deaminase and related PLP enzymes protein sequences. The evolutionary history was inferred by using the Maximum Likelihood method based on the WAG model. A discrete Gamma distribution was used to model evolutionary rate differences among sites (4 categories). Branch support was evaluated using both aLRT (SH like) and bootstrap analysis (100 replicates). Bootstrap values above 0.75 (75%) are displayed in the phylograms shown next to the branches as *. The analysis involved 99 aminoacid sequences and 570 patterns were found (out of a total of 594 sites). Sequences used for the construction of this phylogram are described in Table S6.

To gain additional knowledge regarding the origin and evolution of ACC deaminase multiple searches of the database were conducted; sequences showing a high similarity to different deaminases were obtained and a phylogram was constructed (Fig. 5). In this instance it was observed that ACC deaminase forms a distinct and unique group, where ACC deaminases from different organisms like Bacteria and Fungi cluster together. This is also observed with D-cysteine desulfhydrase, however only a few representatives of the considered “true” D-cysteine desulfhydrases (*E.coli* D-cysteine desulfhydrase) were obtained. When searching in the database, it was observed that D-cysteine desulfhydrase is an enzyme whose distribution is not widespread and it may also be not nearly as conserved as ACC deaminase. Its presence has been verified mainly in γ-Proteobacteria. Other proteins showing some homology were found in Firmicutes and other α-Proteobacteria, but in those instances showing low identity scores (39%).

Interestingly, D-cysteine desulfhydrase activity has been demonstrated for *Solanum lycopersicum* [58] and *Arabidopsis thaliana* [87]. However, these enzymes form a distinct phylogenetic cluster, far away from *E. coli* and other γ-Proteobacteria D-cysteine desulfhydrase. Controversially, ACC deaminase activity has also been demonstrated for *Arabidopsis thaliana*. Although Riemenschneider et al. [87] did not detect ACC deaminase activity from the product of *Arabidopsis thaliana* gene “At1g48420”, McDonell et al. [88] showed that this gene encoded a protein with the ability to breakdown ACC. Moreover, McDonnel et al. [88] suggest that the gene is responsible for regulation of *Arabidopsis thaliana* endogenous ACC levels. The same authors also suggest that this enzyme may be present in many other plant species. Curiously, Todorovic and Glick [58] did not find ACC deaminase activity in the *Solanum lycopersicum* At1g48420 protein homolog, sharing 70% identity and clustering together with *Arabidopsis thaliana* At1g48420 protein (Fig. 5).

Despite showing D-cysteine desulfhydrase and ACC deaminase activity *in vitro*, it is conceivable that the at1g48420 gene product does not represent a “true” D-cysteine desulfhydrase or ACC deaminase, or at least, is only distantly related to bacterial D-cysteine desulfhydrase and ACC deaminase. The grouping that is observed in Fig. 5 supports this latter conclusion. Instead of clustering with bacterial ACC deaminase or D-cysteine desulfhydrase, the plant protein homologs form a distant and unique cluster with a different phylogenetic background within the broader family of these PLP-dependent enzymes.

Proteins with similar origin and function often tend to be conserved. Thus, if the at1g48420 gene encoded a true ACC deaminase (similar phylogenetic background and specialization towards ACC breakdown), it would likely cluster together with bacterial and fungal ACC deaminases and have similar amino acid residues in specific sites as are present in functional ACC deaminases that are important for ACC breakdown (i.e E295, L322). Nevertheless, it is possible that proteins like At1g48420 evolved and specialized in ACC degradation in a different route than those bacterial and fungal ACC deaminases. In this case, additional studies are necessary to further unveil the characteristics of At1g48420 like proteins.

It is most likely that the ability of at1g48420 gene product to use D-cysteine or ACC as substrates results from the high promiscuity that many deaminases show in cleaving multiple substrates that share similar characteristics. For example, it has been shown that ACC deaminase from *Pseudomonas* sp. ACP is able to use multiple substrates like D-cysteine and also other D-aminoacids. *E. coli* and *S. thyphimurium* D-cysteine desulfhydrases are able to efficiently use β-chloro-D-alanine (β-CDA) and other substrates (Table 1).

**Table 1:** Substrate cleavage abilities of studied ACC deaminase, D-cysteine desulfhydrase and other PLP dependent (ACC deaminase or D-cysteine desulfhydrase homologs) enzymes.

Enzyme	Tested substrates	Functional substrates	K_m_ (mM)	Reference
***Arabidopsis thaliana*** **“At1g48420” ACC deaminase homolog**	ACC, D-cys	ACC, D-cys	0.25 (D-cys)	[87], [88]
***Bradyrhizobium japonicum*** **USDA110 ACC deaminase**	ACC, D-ser, β-CDA	ACC, D-ser	n.a	[61]
***Cyberlidnera saturnus*** **ACC deaminase**	ACC, DCA, D-cys, D-ser, β-CDA, OAD-ser	ACC, DCA, D-cys, D-ser	2.6 (ACC)	[1], [102]
***E. coli*** **D-cysteine desulfhydrase**	D-cys, 3-CDA, D-cyst, DLAC, DLSC, DLSCyst, Dlan, D-ala, L-ala, D-ser, L-ser, D-phen, L-phen, D-tryp, and others.	D-cys, 3-CDA, D-cyst, DLac, DLsc, DLSCyst, DLan.	0.15 (D-cys), 0.91 (3-CDA), 0.27 (D-cyst), 0.29 (DLac), 0.04 (DLsc), 0.11 (Dlan)	[8]
***Methylobacterium nodulans*** **ACC deaminase**	ACC, D-cys, L-cys	ACC	0.8 (ACC)	[103]
***Methylobacterium radiotolerans*** **ACC deaminase**	ACC, D-cys, L-cys	ACC	1.8 (ACC)	[103]
***Penicillium citrinum*** **ACC deaminase**	ACC, DCA, L-ser, D-ser, DACC, DACA	ACC, DCA, D-ser	4.8 (ACC)	[65]
***Pirococcus horikoshi*** **OT3 ACC deaminase homolog**	ACC, D-Ala, L-Ala, D- Ser, and L-Ser, D-cys	D-ser, L-ser	n.a	[7]
***Pseudomonas putida*** **UW4 ACC deaminase**	ACC, D-cys	ACC, D-cys	3.4 (ACC)	[104]
***Pseudomonas putida*** **UW4 ACC deaminase mutant E295S/L322T**	ACC, D-cys	D-cys	0.34 (D-cys)	[104]
***Pseudomonas*** **sp. ACP ACC deaminase**	ACC, D-VG, β-CDA, β-FDA, D-ser, V-ACC, APC, Cys, L-hom, L-thr, L-try, L-met, L-tyr, L-cys, L-aba, DCA, D-EAC, D-TAF	ACC, D-VG, β-CDA, D-ser, β-FDA, V-ACC, OAD-ser, β-2CDA, β-2FDA, DCA, D-EAC, D-TAF	1.5 and 9.2 (ACC), 1.1 (β-FDA), 4.4 (V-ACC), 5.4 (β-CDA), 36.2 (DCA), 56 (OAD-ser), 97 (D-VG)	[1], [7], [105]
***Salmonella typhimurium*** **D-cysteine desulfhydrase**	D-Cys, β-CDA, D-Ser, L-Ser, ACC, D-Ala	D-Cys, β-CDA, D-ser	0.34 (D-cys)	[9]
***Solanum lycopersicum*** **ACC deaminase homolog**	ACC, D-cys, L-cys	D-cys	0.21 (D-cys)	[58]
***Solanum lycopersicum*** **ACC deaminase homolog** **S358E/T386L**	ACC, D-cys	ACC	n.a	[58]

1-amino-2-vinylcyclopropane-l-carboxylic acid (**V-ACC**), 1-aminocyclopentane-1-carboxylate (**APC**), 1-aminocyclopropane-1-carboxylic acid (**ACC**), 3-chloro-D-alanine (**3-CDA**), Cystathionine (**Cyst**), D-alanine (**D-ala**), D-cysteine (**D-Cys**), D-cystine (**D-cyst**), D-erythro-2-amino-3-chlorobutyrate (**D-EAC**), D-methionine (**D-met**), D-phenylalanine (**D-phen**), D-serine (**D-ser**), D-threo-2-amine-3-fluorobutyrate (**D-TAF**), D-tryptophan (**D-tryp**), D-vinylglycine (**D-VG**), Dimethyl-ACC (**DACC**), DL-lanthionine (**Dlan**), DL-allocoronamic acid (**DACA**), DL-allocystathionine (**DLAC**), DL-coronamic acid (**DCA**), DL-selenocysteine (**DLSCyst**), DL-selenocystine (**DLSC**), L-alanine (**L-ala**), L-aminobutyric acid (**L-aba**), L-cysteine (**L-cys**), L-homoserine (**L-hom**), L-methione (**L-met**), L-phenylalanine (**L-phen**), L-serine (**L-ser**), L-threonine (**L-thr**), L-tryptophan (**L-try**), L-tyrosine (**L-tyr**), β -chloro-D-alanine (**β-CDA**), β-fluoro-D-alanine (**β-FDA**), β, β- dichloro-D-alanine (**β2CDA**), β, β- difluoro-D-alanine (**β2FDA**), Ο-acetyl-D-serine (**OAD-ser**).

Intriguingly, Todorovic and Glick [58] demonstrated that mutations in amino acids (E295S/L322T) in *Pseudomonas* sp. UW4 ACC deaminase lead to the loss of ACC deaminase activity, yet, these mutations conferred an increased D-cysteine desulfhydrase activity to the mutant enzyme. The Km of the double mutant for D-cysteine was much lower than the Km of the native ACC deaminase towards ACC. Moreover, the Km of the double mutant enzyme towards D-cysteine is equivalent to that of a true D-cysteine desulfhydrase. Still, this mutant enzyme shows great inefficiency (K cat = 10.9 min-1) in D-cysteine cleavage. This data shows that small amino acid changes can confer different substrate usage abilities in closely related deaminases.

If ACC deaminase can use multiple substrates, it is possible that in some organisms the production of ACC deaminase can be important for cleavage of such substances, thus, giving these organisms the ability to use other nutrient sources, or to grow under otherwise toxic conditions. This can have major implications in a microorganism's fitness, especially in organisms living under limiting conditions. For example, Soutourina et al. [89] demonstrated that the expression of D-cysteine desulfhydrase by *E.coli* relieved some of the toxic effects of D-cysteine in bacterial growth. Also, D-cysteine desulfhydrase production allowed the growth of *E. coli* in a minimal medium containing D-cysteine as the sole sulfur source, demonstrating the importance of D-cysteine desulfhydrase in bacterial growth under sulfate limitation. It is possible that by maintaining a broad ability to cleave ACC-like substrates and some D-aminoacids, ACC deaminase genes were maintained in organisms that live in environments where ACC is not present. On the other hand, the presence of ACC deaminase in organisms that are associated with plants or other ACC-producing organisms, gave them a significant advantage in their ecology so that *acdS* genes were maintained. This may have led to the significant *acdS* gene presence in plant associated microorganisms, especially plants grown under perennially stressful conditions [51], and increased *acdS* gene loss in microorganisms living in environments where ACC is nonexistent.

Overall, it seems that bacterial and fungal ACC deaminases (here considered to be the representatives of true ACC deaminases) belong to a large group of PLP dependent deaminases (including bacterial D-cysteine desulfhydrase) related to tryptophan synthase beta subunit and sharing a common origin. Further, mutations and other evolutionary forces may have lead to some level of substrate specialization. Yet, some conserved features appear to allow these enzymes to be able to utilize a wide range of related substrates. This is exemplified by the data presented in Table 1.

### ACC deaminase phylogenetic distribution and evolution

A model for ACC deaminase evolution and phylogenetic distribution is proposed based on the AcdS phylogenetic analysis, AcdS protein sequence analysis, *acdS* gene location, organism habitat and origin. The evolutionary relationship among Archaea, Bacteria and Eukaryotes was also taken into account when attempting to resolve the evolution of ACC deaminase [90], [91], [78].

From the available sequence data, it would appear that the most ancient point for the origin of ACC deaminase in Bacteria dates to the Actinobacteria or Deinococcus-Thermus. Most Actinobacteria strains investigated (Table S1) possess an *acdS* gene in their primary and unique chromosome. In another ancient bacterial lineage, the Deinococcus-Thermus, the *acdS* gene is also found in the chromosome of its representatives *Meiothermus*, suggesting an *acdS* chromosomal location in a common ancestor for Bacteria.

In many α-Proteobacteria, including *Azorhizobium* and *Bradyrhizobium*, the *acdS* gene is found in the primary chromosome of these strains. The *acdS* gene is also found in the primary chromosome of many marine α-Proteobacteria, and in the vinegar isolate *Gluconacetobacter xylinus* NBRC 3288. Despite the fact that ACC deaminase genes were not yet detected in any δ or ε -Proteobacteria (181 genome sequences available in the database), the presence of *acdS* genes in α-Proteobacterial strains that live in environments where ACC is not present, suggests that *acdS* was present in a Proteobacteria ancestor, located in its primary chromosome and likely was acquired by vertical transmission.

Many α-Proteobacteria have *acdS* genes located on plasmids, symbiotic islands or second chromosomes; this is the case of the studied Rhizobiaceae (*Rhizobium*, *Sinorhizobium*, and *Agrobacterium*), Phyllobacteriaceae (*Phyllobacterium* and *Mesorhizobium*) and *Azospirillum* strains (Table S2). Extensive gene transfer analysis between completely sequenced α-Proteobacteria suggested that secondary chromosomes originated from intragenomic transfers from primary chromosomes to ancestral plasmids [92]. This mechanism may have not only led to the origin of a second chromosome in some α-Proteobacteria but also in other Proteobacteria. In this regard, it is possible that *acdS* was transferred from the primary chromosome to a plasmid in some α-Proteobacteria. This possibility is consistent with the presence of *acdS* genes in the plasmids of *Rhizobium* and *Sinorhizobium* species and in the second chromosome of *Rhizobium radiobacter* K84 and *Agrobacterium vitis* S4. Slater et al. [92] also suggested that some strains like *Mesorhizobium* could have evolved by plasmid gene integration into the main chromosome. This suggestion is consistent with the observation that the same gene arrangement is found between *Mesorhizobium* symbiotic islands and some *Rhizobium* and *Sinorhizobium* symbiotic plasmids, where the *acdS* gene is located.

It is likely that intragenomic transfers of *acdS* genes from primary chromosomes to plasmids may have occurred in members of α-Proteobacteria as well as in β and γ-Proteobacteria. The presence of *acdS* genes in the second chromosome of *Burkholderia* and megaplasmids in *Ralstonia* and some strains of *Pseudomonas* is consistent with this idea. The occurrence of such phenomena may have led to a dispersal of *acdS* genes through plasmids that are readily transmissible between closely and more distant related strains. This leads to the puzzling phylogeny of the *acdS* gene that is observed in bacteria belonging to the same Order or Family (taxonomy) (Fig. 1).

In most Fungi, AcdS sequences share an average sequence identity of ∼50% with Bacterial AcdS. An exception to this case is the AcdS from yeasts, *Punctularia strigosozonata* HHB-11173, *Fomitopsis pinicola* FP-58527 (Basidiomycota/Agaricomycetes), *Aureobasidium pullulans* AY4, *Macrophomina phaseolina* MS6 and *Guignardia citricarpa* CGMCC3.14348 (Ascomycota/Dothideomycetes), and Stramenopiles, which appear to have a Bacterial origin. As observed in the AcdS based phylogram (Fig. 3), it seems that Fungal (excluding the above mentioned exceptions) and Bacterial AcdS diverged long ago. At this point it's not possible to corroborate both hypothesis of AcdS monophyletic or paraphyletic origin. Still, protein sequence analysis show some conserved amino acid regions (e.g. His80 and Ala161) in ACC deaminase from Fungi, Actinobacteria, Deinococcus-Thermus, and α-Proteobacteria, suggesting a common origin for *acdS* in these organisms. Organisms belonging to β- and γ-Proteobacterial classes show different amino acids in the referred positions, suggesting a later divergence from the α-Proteobacteria and the rest of ancient classes. The Fungi grouping closer to Actinobacteria is also observed in the phylogram (Fig. 3) suggesting a common origin for ACC deaminase in these organisms.

Based on the currently available data, we suggest that *acdS* genes had an ancient origin that may date to a Eukaryote and Bacterial common ancestor that possessed this gene in its chromosome. Furthermore, it is most likely that ACC deaminase originated as a consequence of specific mutations in an already existing PLP dependent enzyme showing high similarity to tryptophan synthase beta subunit. This is consistent with the results obtained by Todorovic and Glick [58] showing that small amino acid changes in related enzymes can be responsible for the ability to use a specific substrate.

Through time, it is probable that the *acdS* gene evolved by continuous vertical transmission, in which different constraints like habitat adaptation led to *acdS* divergence and sometimes gene loss. Intragenomic transfers of *acdS* genes from primary chromosomes to plasmids may have been selected for as a consequence of the advantage of ACC deaminase production, and this probably led to HGT events and increased divergence of *acdS* genes. These intragenomic transfer events and the presence of *acdS* on plasmids may have also lead to gene loss in many organisms. This is consistent with the results obtained by Prigent-Combaret et al. [40] showing that *Azospirillum lipoferum* 4B loses the plasmid containing an *acdS* gene during phenotypic variation events.

### The role of ACC deaminase production in microorganism's ecology and fitness

From the available information, it is observed that many of the *acdS*+ organisms here described were isolated from heavily contaminated soils or otherwise stressed environments (Tables S1–S5), suggesting that ACC deaminase-producing microorganisms are more prevalent and better able to live in such conditions.

Organisms that produce ACC deaminase normally bind to plant tissues, and take up ACC to convert into ammonia and α-ketobutyrate [2]. The products of ACC cleavage are potential nitrogen and carbon sources [2], [33] that can play a role in the microorganism's fitness under stressful situations. Under stress conditions plants produce higher levels of the phytohormone ethylene, which means that the plants also produce higher levels of ACC [3]. Microorganisms that bind to plant tissues typically utilize plant exudates as a nutrient source. Under stress conditions, not only is the amount of ACC produced by the plant increased, the vast majority of rhizosphere microorganisms produce the phytohormone indoleacetic acid (IAA) which acts to loosen plant cell walls thereby facilitating root exudation. Bacterial IAA production has also been shown to increase ACC synthase expression in plants [93]. Thus, microorganisms that can both produce IAA and utilize ACC may have a competitive advantage over other soil microorganisms [2], [94].

Importantly, a recent study by Timmusk et al. [51], showed that ACC deaminase-producing organisms were more much abundant in the rhizosphere of wild barley (*Hordeum spontaneum*) growing in a stressed environment than they were in a similar (nearby) less stressed environment. This result was obtained despite the fact that both environments had similar soil, rock and topology characteristics. In addition, ACC deaminase-producing bacteria were abundant in plant rhizosphere samples and almost nonexistent in bulk soil samples. This suggests that organisms that produce ACC deaminase more readily survive in stressed environments by the mutualistic interaction with a plant host.

By degrading ACC, microorganisms decrease plant ethylene levels that under stress conditions are responsible for plant senescence and ultimately plant death [3]. Therefore, these organisms facilitate plant health under stress conditions. In turn, healthier plants provide their associated microorganisms with more nutrients thereby increasing the proliferation of these microorganisms.

Chen et al. [95] demonstrated that ACC deaminase-producing bacteria are also present in the casing soil of the ethylene-producing fungi *Agaricus bisporus*. The authors proposed a new model for the interaction between fungi and ACC deaminase producing bacteria. Bacteria possessing an *acdS* gene were able to increase fungal primordium initiation and proliferation by reducing endogenous ACC levels and consequently the inhibitory ethylene levels known to affect fungal development. These results show that ACC deaminase-producing bacteria might not only associate with plants but also with fungi, bringing significant advantages to fungal colonization in soil. On the other hand, bacteria producing ACC deaminase gain significant advantages by associating with extreme soil and plant colonizers like fungi. Being that these organisms constantly produce ACC, bacteria able to degrade ACC may gain extra nutrient sources as previously suggested.

### ACC deaminase in Fungi: Relationship with plants or regulation of endogenous ACC levels?

The production of ACC deaminase by *Trichoderma asperellum* T230 has been shown to be an important mechanism for the plant growth promotion abilities of this fungal strain [16]. When ACC deaminase production is impaired, the fungal ability to promote canola root elongation is decreased, therefore, suggesting that ACC deaminase may act in a similar way as previously described by Glick et al. [2] for plant growth-promoting bacteria.

Nonetheless, it has been shown by Jia et al. [65] that ACC deaminase in *Penicillium citrinum* is produced independently of a relationship with a plant host. This happens because *Pennicillium citrinum* is capable of producing and accumulating ACC in its tissues. That is, *Penicillium citrinum* possesses not only an *acdS* gene but also an ACC synthase gene. Jia et al. [96] found that the ACC deaminase was induced by the presence of accumulated ACC in the intracellular spaces of *Penicilium citrinum*, indicating that ACC deaminase may participate in the regulation of ACC levels in this strain. As a consequence, ethylene production by *P. citrinum* can also be regulated by ACC deaminase. In fact, our search of the database revealed the presence of ACC synthase homologs in most fungal strains that possess an ACC deaminase (data not shown). Together these results suggest that ACC deaminase production by Fungi can account for the regulation of endogenous ACC concentrations, and therefore regulation of ethylene levels which can inhibit primordium initiation and formation.

### What is the role of ACC deaminase in pathogenic microorganisms?

Surprisingly, *acdS* and *acdR* genes are found in a wide range of plant and human pathogenic microorganisms (Tables S1-S5), suggesting that ACC deaminase may play a role in these microrganisms' ecology. For example, the production of ACC deaminase has been reported in the human pathogenic *Burkholderia cenocepacia* J2315 [64]. However, this bacterial strain, like other pathogenic *Burkholderia* strains, is predominant in soils where it normally associates with plants [97]–[99]. The *acdS* gene is also found in pathogenic fungi like *Aspergillus* spp. and *Myceliophthora thermophila*. Despite causing severe diseases in immunocompromised humans, these strains are mainly found in soil [100], [101]. This data suggests that the presence of *acdS* genes in human pathogenic organisms may not be related to their human pathogenesis mechanisms but rather to their possible ecological role in soil. Also, it is possible that the presence of *acdS* gene in these strains and in plant pathogenic bacteria is related to the continuous *acdS* vertical transmission and not to any beneficial effects of ACC deaminase production.

Nevertheless, ACC deaminase production by pathogenic microorganisms may ultimately play a role in: (a) obtaining extra nutrients sources from ACC or ACC like substrate degradation, (b) the plant or fungi growth promoting abilities of these organisms when they are not acting as human or plant pathogens (“opportunistic” pathogens), (c) augmenting the ability to overcome ethylene or ACC mediated plant response systems, (d) regulation of endogenous ACC levels, or a combination of these factors.

## Conclusions

The results obtained in this study provide a more complete view of the role for ACC deaminase-producing organisms then was previously available. ACC deaminase genes are not only found in plant-associated microorganisms but also in other bacterial and fungal strains isolated from a wide range of different sources (i.e. hot springs, industrial sludge, sea), hence, challenging the notion that ACC deaminase-producing organisms only interact with plants, or more interestingly, that ACC deaminase can only use ACC as a substrate.

Based on multiple parameters like protein sequence analysis and phylogenetic studies we suggest that ACC deaminase belongs to a broad group of promiscuous PLP dependent enzymes (tryptophan synthase beta subunit family) sharing a common ancestor. It is most likely that ACC deaminase originated as a consequence of specific mutations in its ancestral enzyme gene. Small amino acid mutations conferred changes in substrate specificity, however, the ability to degrade similar substrates was somehow maintained. This can account for the presence of *acdS* genes in bacteria that don't associate with ACC producing organisms. The continuous vertical transmission of *acdS* genes may also be responsible for the presence of *acdS* in these organisms. Furthermore, contrary to previous reports, here we demonstrate that the *acdS* gene is mostly vertically inherited in various bacterial and fungal classes. An ancient origin dating a Bacterial/Eukaryote ancestor is also proposed for the *acdS* gene.

Nonetheless, horizontal gene transfer does account for a wide portion of ACC deaminase evolution. For instance, some fungal classes and some members of Stramenopiles may have acquired *acdS* genes from Bacteria, suggesting that HGT events not only occur between bacteria but also may occur between distantly related organisms.

The presence of the *acdR* gene is observed in most Proteobacteria possessing an *acdS* gene, suggesting a coupled evolution for these genes. In other microorganisms like Actinobacteria and Deinococcus-Thermus (*Meiothermus*) the presence of genes encoding a GntR family protein are observed in the vicinity of the *acdS* gene, suggesting a different mechanism of ACC deaminase regulation. Moreover, these regulatory genes (here termed *acd-AR* and *acd-MR*) are mostly found in these *acdS*+ bacteria groups, reinforcing the idea that specific regulatory elements can be found in different Bacteria classes.

Additional genetic and biochemical studies are needed to gain some additional understanding of ACC deaminase functioning and its possible role(s) in the ecology of various organisms. Also, exploring the origin of ACC deaminase and related enzymes may bring new insights into the functioning of this PLP family of enzymes that may be the key to their use in a variety of important biotechnological applications.

## Supporting Information

Figure S1 **Multiple sequence alignment based on functional ACC deaminases, putative ACC deaminase sequences from** ***Agreia*** **sp. PHSC20C1,** ***Rhodococcus*** **sp. R04 (Actinobacteria) and** ***Meiothermus ruber*** **DSM1279 (Deinococcus-Thermus).** D-cysteine desulfhydrase from *E-coli*, PLP dependent deaminase from *Pyrococcus horikoshii* and PLP dependent deaminase from *Solanum lycopersicum* are highlighted in grey. Conserved residues between all protein groups are shown in blue. ACC deaminase conserved residues are shown in green.
(PDF)

Figure S2 Putative regulators, *acdS* and neighborhood genes organization in some Actinobacteria, Deinococcus-Thermus and Proteobacteria.
(PDF)

File S1 **Evolutionary distances estimates of** ***acdS*** **and 16S rRNA genes from several bacterial groups.**
(XLSX)

Table S1 **Accession numbers for Actinobacteria, Deinococcus-Thermus and Firmicutes 16S rRNA,** ***acdS*** **and** ***acdR*** **genes and AcdS and AcdR protein sequences.** Description of the *acdS* gene location, ACC deaminase (ACCD) activity, strains relative habitat and geographical origin.
(DOCX)

Table S2 **Accession numbers for α-Proteobacteria 16S rRNA,** ***acdS*** **and** ***acdR*** **genes and AcdS and AcdR proteins sequences.** Description of the *acdS* gene location, ACC deaminase (ACCD) activity, strains relative habitat and origin.
(DOCX)

Table S3 **Accession numbers for β-Proteobacteria 16S rRNA,** ***acdS*** **and** ***acdR*** **genes and AcdS and AcdR proteins sequences and description of the** ***acdS*** **gene location, ACC deaminase (ACCD) activity, strains relative habitat and origin.**
(DOCX)

Table S4 **Accession numbers for γ-Proteobacteria 16S rRNA,** ***acdS*** **and** ***acdR*** **genes and AcdS and AcdR proteins sequences and description of the** ***acdS*** **gene location, ACC deaminase (ACCD) activity, strains relative habitat and origin.**
(DOCX)

Table S5 **Accession numbers for Eukaryotes AcdS complete sequences and description of ACC deaminase (ACCD) activity, strains relative habitat and geographical origin.**
(DOCX)

Table S6 **Acession numbers for the sequences used in** **Figure 5** **.**
(DOCX)
