# Supplementary material for: New Insights into 1-Aminocyclopropane-1-Carboxylate (ACC) Deaminase Phylogeny, Evolution and Ecological Significance
Source: PLoS One. 2014 Jun 6;9(6):e99168. doi: 10.1371/journal.pone.0099168 (PMC4048297; doi:10.1371/journal.pone.0099168)
Supplement: Figure S1 — Multiple sequence alignment based on functional ACC deaminases, putative ACC deaminase sequences from Agreia sp. PHSC20C1, Rhodococcus sp. R04 (Actinobacteria) and Meiothermus ruber DSM1279 (Deinococcus-Thermus). D-cysteine desulfhydrase from E-coli, PLP dependent deaminase from Pyrococcus horikoshii and PLP dependent deaminase from Solanum lycopersicum are highlighted in grey. Conserved residues between all protein groups are shown in blue. ACC deaminase conserved residues are shown in green. (PDF) [file pone.0099168.s001.pdf]

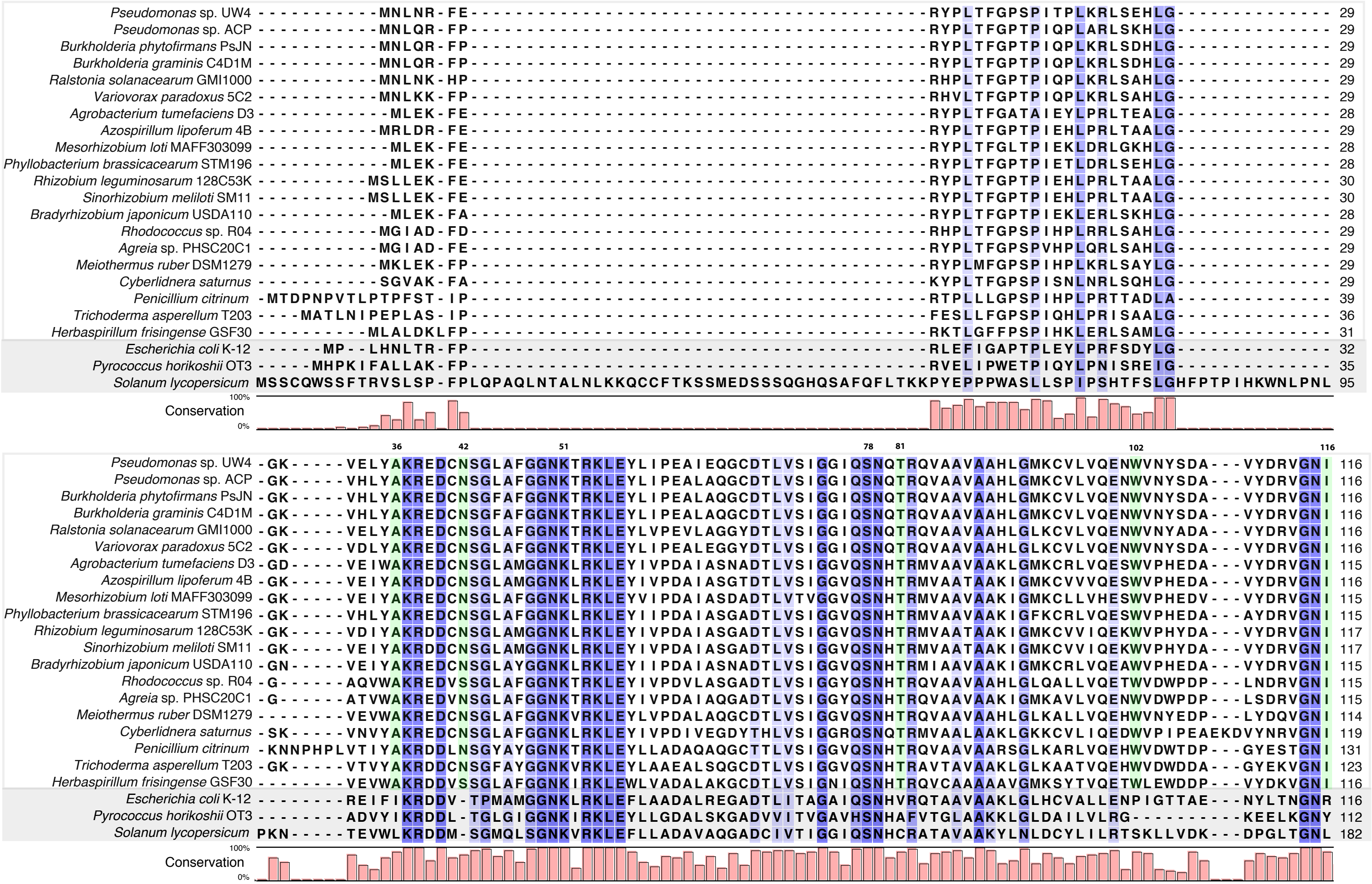

Pseudomonas sp. UW4
Pseudomonas sp. ACP
Burkholderia phytofirmans PsJN
Burkholderia graminis C4D1M
Ralstonia solanacearum GMI1000
Variovorax paradoxus 5C2
Agrobacterium tumefaciens D3
Azospirillum lipoferum 4B
Mesorhizobium loti MAFF303099
Phyllobacterium brassicacearum STM196
Rhizobium leguminosarum 128C53K
Sinorhizobium meliloti SM11
Bradyrhizobium japonicum USDA110
Rhodococcus sp. R04
Agreia sp. PHSC20C1
Meiothermus ruber DSM1279
Cyberlindnera saturnus
Penicillium citrinum
Trichoderma asperellum T203
Herbaspirillum frisingense GSF30
Escherichia coli K-12
Pyrococcus horikoshii OT3
Solanum lycopersicum
Conservation
100%
0%
36
42
51
78
81
102
116

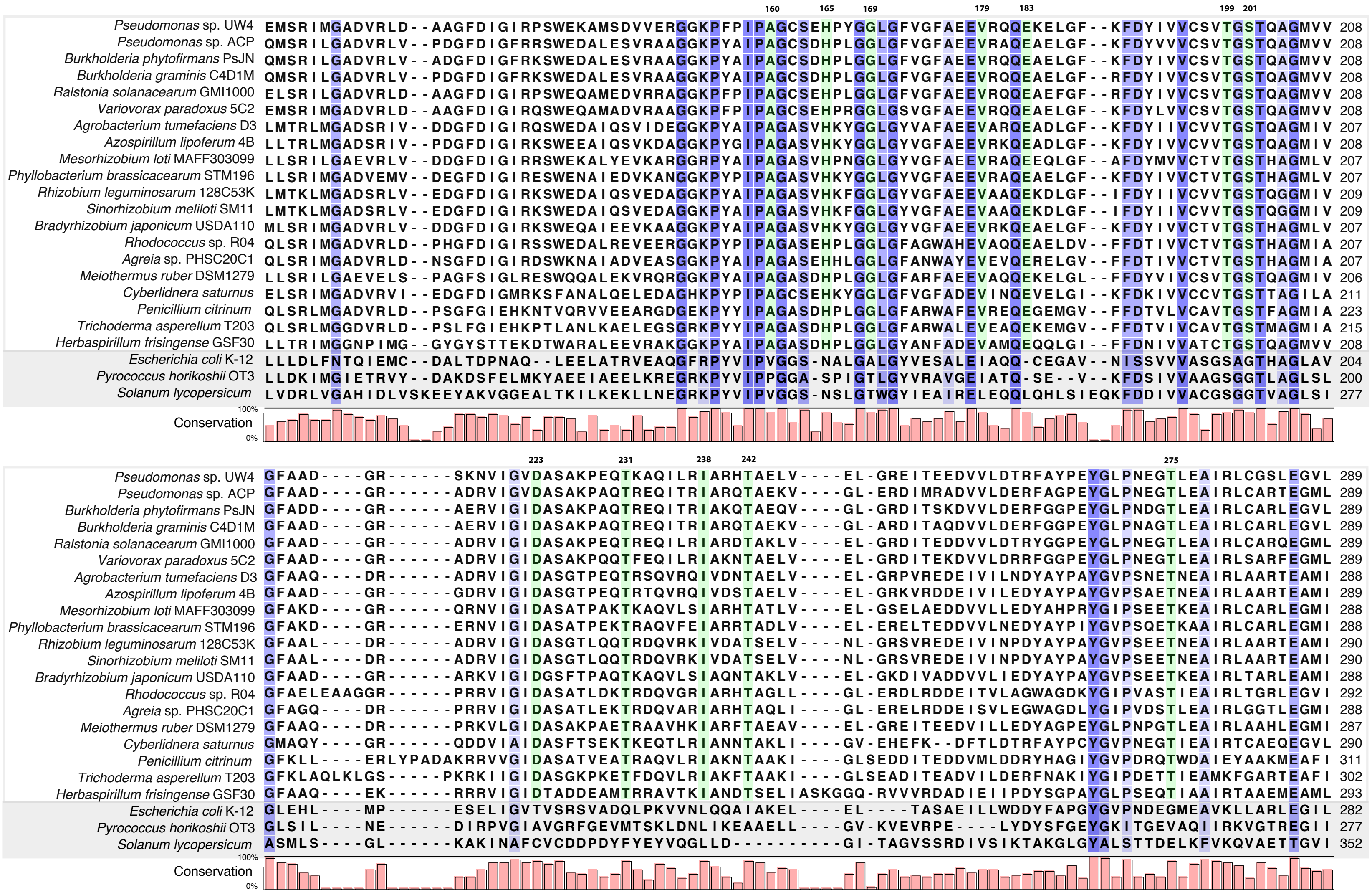
160 165 169 179 183 199 201
Pseudomonas sp. UW4 EMSRIMGADVRLD--AAGFDIGIRPSWEKAMSDVVERGGKPFPIPAGCSEHPYGGLGFVGFAEEVRQQEKELGF--KFDYIVVCSVTGSTQAGMVV 208
Pseudomonas sp. ACP QMSRILGADVRLV--PDGFDIGFRRSWEDALESVRAAGGKPYAIPAGCSDHPLGGLGFVGFAEEVRAQEAELGF--KFDYVVVCSVTGSTQAGMVV 208
Burkholderia phytofirmans PsJN QMSRILGADVRLV--ADGFDIGFRKSWEDALESVRAAGGKPYAIPAGCSDHPLGGLGFVGFAEEVRQQEAELGF--KFDYIVVCSVTGSTQAGMVV 208
Burkholderia graminis C4D1M QMSRILGADVRLV--PDGFDIGFRKSWEDALESVRAAGGKPYAIPAGCSDHPLGGLGFVGFAEEVRQQEAELGF--RFDYIVVCSVTGSTQAGMVV 208
Ralstonia solanacearum GMI1000 ELSRILGADVRLD--AAGFDIGIRPSWEQAMEDVRRAGGKPFPIPAGCSEHPLGGLGFVGFAEEVRQQEAEFGF--RFDYIVVCSVTGSTQAGMVV 208
Variovorax paradoxus 5C2 EMSRIMGADVRLD--AAGFDIGIRQSWEQAMADVRAAGGKPFPIPAGCSEHPRGGLGSVGFAEEVRQQEAELGF--KFDYLVVCSVTGSTQAGMVV 208
Agrobacterium tumefaciens D3 LMTRLMGADSRIV--DDGFDIGIRQSWEDAIQSVIDEGGKPYAIPAGASVHKYGGLGYVAFAEEVARQEADLGF--KFDYIIVCVVTGSTQAGMIV 207
Azospirillum lipoferum 4B LLTRLMGADSRIV--PDGFDIGIRKSWEEAIQSVKDAGGKPYGIPAGASVHKYGGLGYVGFAEEVRKQEADLGF--KFDYIVVCVVTGSTQAGMIV 208
Mesorhizobium loti MAFF303099 LLSRILGAEVRLV--DDGFDIGIRRSWEKALYEVKARGGRPYAIPAGASVHPNGGLGYVGFAEEVRAQEEQLGF--AFDYMVVCTVTGSTHAGMLV 207
Phyllobacterium brassicacearum STM196 LLSRIMGADVEMV--DEGFDIGIRESWENAIEDVKANGGKPYPIPAGASVHKYGGLGYVGFAEEVRAQEAELGF--KFDYIVVCTVTGSTHAGMLV 207
Rhizobium leguminosarum 128C53K LMTKLMGADSRLV--EDGFDIGIRKSWEDAIQSVEDAGGKPYAIPAGASVHKFGGLGYVGFAEEVAAQEKDLGF--IFDYIIVCVVTGSTQGGMIV 209
Sinorhizobium meliloti SM11 LMTKLMGADSRLV--EDGFDIGIRKSWEDAIQSVEDAGGKPYAIPAGASVHKFGGLGYVGFAEEVAAQEKDLGF--IFDYIIVCVVTGSTQGGMIV 209
Bradyrhizobium japonicum USDA110 MLSRIMGADVRLV--DDGFDIGIRKSWEQAIEEVKAAGGKPYAIPAGASVHKYGGLGYVGFAEEVRKQEAELGF--KFDYIVVCTVTGSTHAGMLV 207
Rhodococcus sp. R04 QLSRIMGADVRLD--PHGFDIGIRSSWEDALREVEERGGKPYPIPAGASEHPLGGLGFAGWAHEVAQQEAELDV--FFDTIVVCTVTGSTHAGMIA 207
Agreia sp. PHSC20C1 QLSRIMGADVRLD--NSGFDIGIRDSWKNAIADVEASGGKPYAIPAGASEHHLGGLGFANWAYEVEVQERELGV--FFDTIVVCTVTGSTHAGMIA 207
Meiothermus ruber DSM1279 LLSRILGAEVELS--PAGFSIGLRESWQQALEKVRQRGGKPYAIPAGASDHPLGGLGFARFAEEVAQQEKELGL--FFDYVIVCSVTGSTQAGMIV 206
Cyberlindnera saturnus ELSRIMGADVRVI--EDGFDIGMRKSFANALQELEDAGHKPYPIPAGCSEHKYGGLGFVGFADEVINQEVELGI--KFDKIVVCCVTGSTTAGILA 211
Penicillium citrinum QLSRLMGADVRLD--PSGFGIEHKNTVQRVVEEARGDGEKPYYIPAGASDHPLGGLGFARWAFEVREQEGEMGV--FFDTVLVCAVTGSTFAGMIA 223
Trichoderma asperellum T203 QLSRLMGGDVRLD--PSLFGIEHKPTLANLKAELEGSGRKPYYIPAGASDHPLGGLGFARWALEVEAQEKEMGV--FFDTVIVCAVTGSTMAGMIA 215
Herbaspirillum frisingense GSF30 LLTRIMGGNPIMG--GYGYSTTEKDTWARALEEVRAKGGKPYAIPAGASDHPLGGLGYANFADEVAMQEQQLGI--FFDNIVVATCTGSTQAGMVV 208
Escherichia coli K-12 LLLDLFNTQIEMC--DALTDPNAQ--LEELATRVEAQGFRPYVIPVGGS-NALGALGYVESALEIAQQ-CEGAV--NISSVVVASGSAGTHAGLAV 204
Pyrococcus horikoshii OT3 LLDKIMGIETRVY--DAKDSFELMKYAEEIAEELKREGRKPYVIPPGGA-SPIGTLGYVRAVGEIATQ-SE--V--KFDSIVVAAGSGGTLAGLSL 200
Solanum lycopersicum LVDRLVGAHIDLVSKEEYAKVGGEALTKILKEKLLNEGRKPYVIPVGGS-NSLGTWGYIEAIRELEQQLQHLSIEQKFDDIVVACGSGGTVAGLSI 277
Conservation 100% 0%
223 231 238 242 275
Pseudomonas sp. UW4 GFAAD----GR------SKNVIGVDASAKPEQTKAQILRIARHTAELV----EL-GREITEEDVVLDTRFAYPEYGLPNEGTLEAIRLCGSLEGVL 289
Pseudomonas sp. ACP GFAAD----GR------ADRVIGVDASAKPAQTREQITRIARQTAEKV----GL-ERDIMRADVVLDERFAGPEYGLPNEGTLEAIRLCARTEGML 289
Burkholderia phytofirmans PsJN GFADD----GR------AERVIGIDASAKPAQTREQITRIAKQTAEQV----GL-GRDITSKDVVLDERFGGPEYGLPNDGTLEAIRLCARLEGVL 289
Burkholderia graminis C4D1M GFAAD----GR------AERVIGIDASAKPAQTREQITRIARQTAEKV----GL-ARDITAQDVVLDERFGGPEYGLPNAGTLEAIRLCARLEGVL 289
Ralstonia solanacearum GMI1000 GFAAD----GR------ADRVIGIDASAKPEQTREQILRIARDTAKLV----EL-GRDITEDDVVLDTRYGGPEYGLPNEGTLEAIRLCARQEGML 289
Variovorax paradoxus 5C2 GFAAD----GR------ADRVIGIDASAKPQQTFEQILRIAKNTAELV----EL-GRDITEKDVVLDRRFGGPEYGLPNEGTLEAIRLSARFEGML 289
Agrobacterium tumefaciens D3 GFAAQ----DR------ADRVIGIDASGTPEQTRSQVRQIVDNTAELV----EL-GRPVREDEIVILNDYAYPAYGVPSNETNEAIRLAARTEAMI 288
Azospirillum lipoferum 4B GFAAD----GR------GDRVIGIDASGTPEQTRTQVRQIVDSTAELV----EL-GRKVRDDEIVILEDYAYPAYGVPSAETNEAIRLAARTEAMI 289
Mesorhizobium loti MAFF303099 GFAKD----GR------QRNVIGIDASATPAKTKAQVLSIARHTATLV----EL-GSELAEDDVVLLEDYAHPRYGIPSEETKEAIRLCARLEGMI 288
Phyllobacterium brassicacearum STM196 GFAKD----GR------ERNVIGIDASATPEKTRAQVFEIARRTADLV----EL-ERELTEDDVVLNEDYAYPIYGVPSQETKAAIRLCARLEGMI 288
Rhizobium leguminosarum 128C53K GFAAL----DR------ADRVIGIDASGTLQQTRDQVRKIVDATSELV----NL-GRSVREDEIVINPDYAYPAYGVPSEETNEAIRLAARTEAMI 290
Sinorhizobium meliloti SM11 GFAAL----DR------ADRVIGIDASGTLQQTRDQVRKIVDATSELV----NL-GRSVREDEIVINPDYAYPAYGVPSEETNEAIRLAARTEAMI 290
Bradyrhizobium japonicum USDA110 GFAAD----GR------ARKVIGIDGSFTPAQTKAQVLSIAQNTAKLV----EL-GKDIVADDVVLIEDYAYPAYGVPSEETKEAIRLTARLEAMI 288
Rhodococcus sp. R04 GFAELEAAGGR------PRRVIGIDASATLDKTRDQVGRIARHTAGLL----GL-ERDLRDDEITVLAGWAGDKYGIPVASTIEAIRLTGRLEGVI 292
Agreia sp. PHSC20C1 GFAGQ----DR------PRRVIGIDASATLEKTRDQVARIARHTAQLI----GL-ERELRDDEISVLEGWAGDLYGIPVDSTLEAIRLGGTLEGMI 288
Meiothermus ruber DSM1279 GFAAQ----DR------PRKVLGIDASAKPAETRAAVHKIARFTAEAV----EL-GREITEEDVILLEDYAGPEYGLPNPGTLEAIRLAAHLEGMI 287
Cyberlindnera saturnus GMAQY----GR------QDDVIAIDASFTSEKTKEQTLRIANNTAKLI----GV-EHEFK--DFTLDTRFAYPCYGVPNEGTIEAIRTCAEQEGVL 290
Penicillium citrinum GFKLL----ERLYPADAKRRVVGIDASATVEATRAQVLRIAKNTAAKI----GLSEDDITEDDVMLDDRYHAGIYGVPDRQTWDAIEYAAKMEAFI 311
Trichoderma asperellum T203 GFKLAQLKLGS-----PKRKIIGIDASGKPKETFDQVLRIAKFTAAKI----GLSEADITEADVLIDERFNAKIYGIPDETTIEAMKFGARTEAFI 302
Herbaspirillum frisingense GSF30 GFAAQ----EK------RRRVIGIDTADDEAMTRRAVTKIANDTSELIASKGGQ-RVVVRDADIEIIPDYSGPAYGLPSEQTIAAIRTAAEMEAML 293
Escherichia coli K-12 GLEHL----MP------ESELIGVTVSRSVADQLPKVVNLQQAIAKEL----EL----TASAEILLWDDYFAPGYGVPNDEGMEAVKLLARLEGIL 282
Pyrococcus horikoshii OT3 GLSIL----NE------DIRPVGIAVGRFGEVMTSKLDNLIKEAAELL----GV-KVEVRPE----LYDYSFGEYGKITGEVAQIIRKVGTREGII 277
Solanum lycopersicum ASMLS----GL------KAKINAFCVCDDPDYFYEYVQGLLD----------GI-TAGVSSRDIVSIKTAKGLGYALSTTDELKFVKQVAETTGVI 352
Conservation 100% 0%

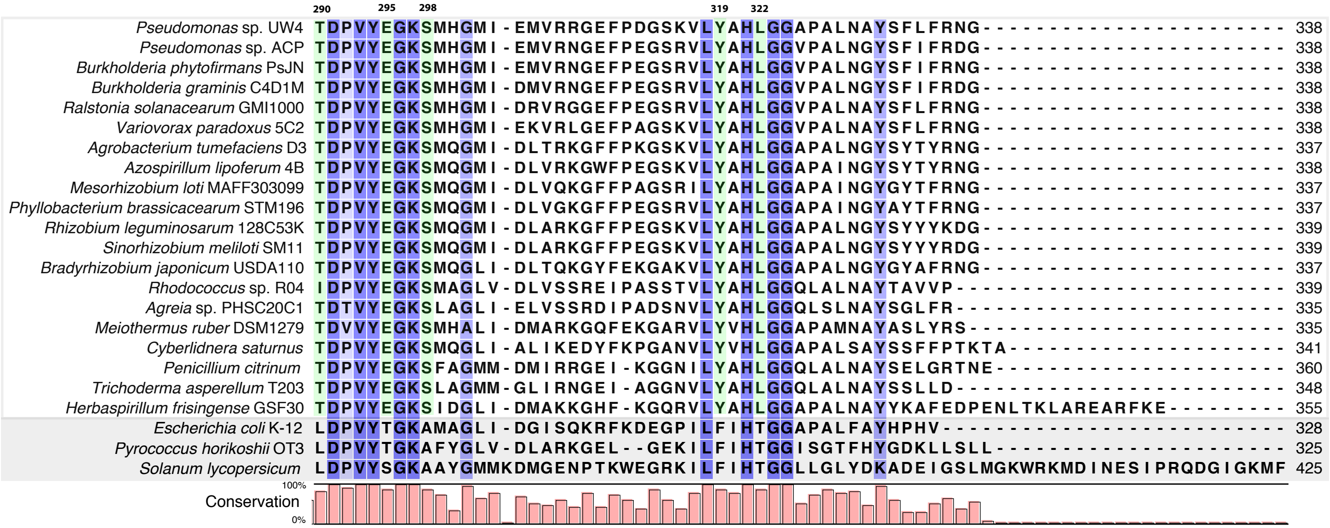
290 295 298 319 322
Pseudomonas sp. UW4 TDPVYEGKSMHGMI-EMVRRGEFPDGSKVLYAHLGGAPALNAYSFLFRNG--------------------- 338
Pseudomonas sp. ACP TDPVYEGKSMHGMI-EMVRNGEFPEGSRVLYAHLGGVPALNGYSFIFRDG--------------------- 338
Burkholderia phytofirmans PsJN TDPVYEGKSMHGMI-EMVRNGEFPEGSRVLYAHLGGVPALNGYSFIFRNG--------------------- 338
Burkholderia graminis C4D1M TDPVYEGKSMHGMI-DMVRNGEFPEGSRVLYAHLGGVPALNGYSFIFRDG--------------------- 338
Ralstonia solanacearum GMI1000 TDPVYEGKSMHGMI-DRVRGGEFPEGSRVLYAHLGGVPALNAYSFLFRNG--------------------- 338
Variovorax paradoxus 5C2 TDPVYEGKSMHGMI-EKVRLGEFPAGSKVLYAHLGGVPALNAYSFLFRNG--------------------- 338
Agrobacterium tumefaciens D3 TDPVYEGKSMQGMI-DLTRKGFFPKGSKVLYAHLGGAPALNGYSYTYRNG--------------------- 337
Azospirillum lipoferum 4B TDPVYEGKSMQGMI-DLVRKGWFPEGSKVLYAHLGGAPAINGYSYTYRNG--------------------- 338
Mesorhizobium loti MAFF303099 TDPVYEGKSMQGMI-DLVQKGFFPAGSRILYAHLGGAPAINGYGYTFRNG--------------------- 337
Phyllobacterium brassicacearum STM196 TDPVYEGKSMQGMI-DLVGKGFFPEGSRVLYAHLGGAPAINGYAYTFRNG--------------------- 337
Rhizobium leguminosarum 128C53K TDPVYEGKSMQGMI-DLARKGFFPEGSKVLYAHLGGAPALNGYSYYYKDG--------------------- 339
Sinorhizobium meliloti SM11 TDPVYEGKSMQGMI-DLARKGFFPEGSKVLYAHLGGAPALNGYSYYYRDG--------------------- 339
Bradyrhizobium japonicum USDA110 TDPVYEGKSMQGLI-DLTQKGYFEKGAKVLYAHLGGAPALNGYGYAFRNG--------------------- 337
Rhodococcus sp. R04 IDPVYEGKSMAGLV-DLVSSREIPASSTVLYAHLGGQLALNAYTAVVP------------------------ 339
Agreia sp. PHSC20C1 TDTVYEGKSLAGLI-ELVSSRDIPADSNVLYAHLGGQLSLNAYSGLFR------------------------ 335
Meiothermus ruber DSM1279 TDVVYEGKSMHALI-DMARKGQFEKGARVLYVHLGGAPAMNAYASLYRS----------------------- 335
Cyberlindnera saturnus TDPVYEGKSMQGLI-ALIKEDYFKPGANVLYVHLGGAPALSAYSSFFPTKTA-------------------- 341
Penicillium citrinum TDPVYEGKSFAGMM-DMIRRGEI-KGGNILYAHLGGQLALNAYSELGRTNE--------------------- 360
Trichoderma asperellum T203 TDPVYEGKSLAGMM-GLIRNGEI-AGGNVLYAHLGGQLALNAYSSLLD------------------------ 348
Herbaspirillum frisingense GSF30 TDPVYEGKSIDGLI-DMAKKGHF-KGQRVLYAHLGGAPALNAYYKAFEDPENLTKLAREARFKE--------- 355
Escherichia coli K-12 LDPVYTGKAMAGLI-DGISQKRFKDEGPILFIHTGGAPALFAYHPHV------------------------- 328
Pyrococcus horikoshii OT3 LDPVYTGKAFYGLV-DLARKGEL--GEKILFIHTGGISGTFHYGDKLLSLL--------------------- 325
Solanum lycopersicum LDPVYSGKAAYGMMKDMGENPTKWEGRKILFIHTGGLLGLYDKADEIGSLMGKWRKMDINESIPRQDGIGKMF 425
100%
Conservation
0%
