# Supplementary material for: New Insights into 1-Aminocyclopropane-1-Carboxylate (ACC) Deaminase Phylogeny, Evolution and Ecological Significance
Source: PLoS One. 2014 Jun 6;9(6):e99168. doi: 10.1371/journal.pone.0099168 (PMC4048297; doi:10.1371/journal.pone.0099168)
Supplement: Figure S2 — Putative regulators, acdS and neighborhood genes organization in some Actinobacteria, Deinococcus-Thermus and Proteobacteria. (PDF) [file pone.0099168.s002.pdf]

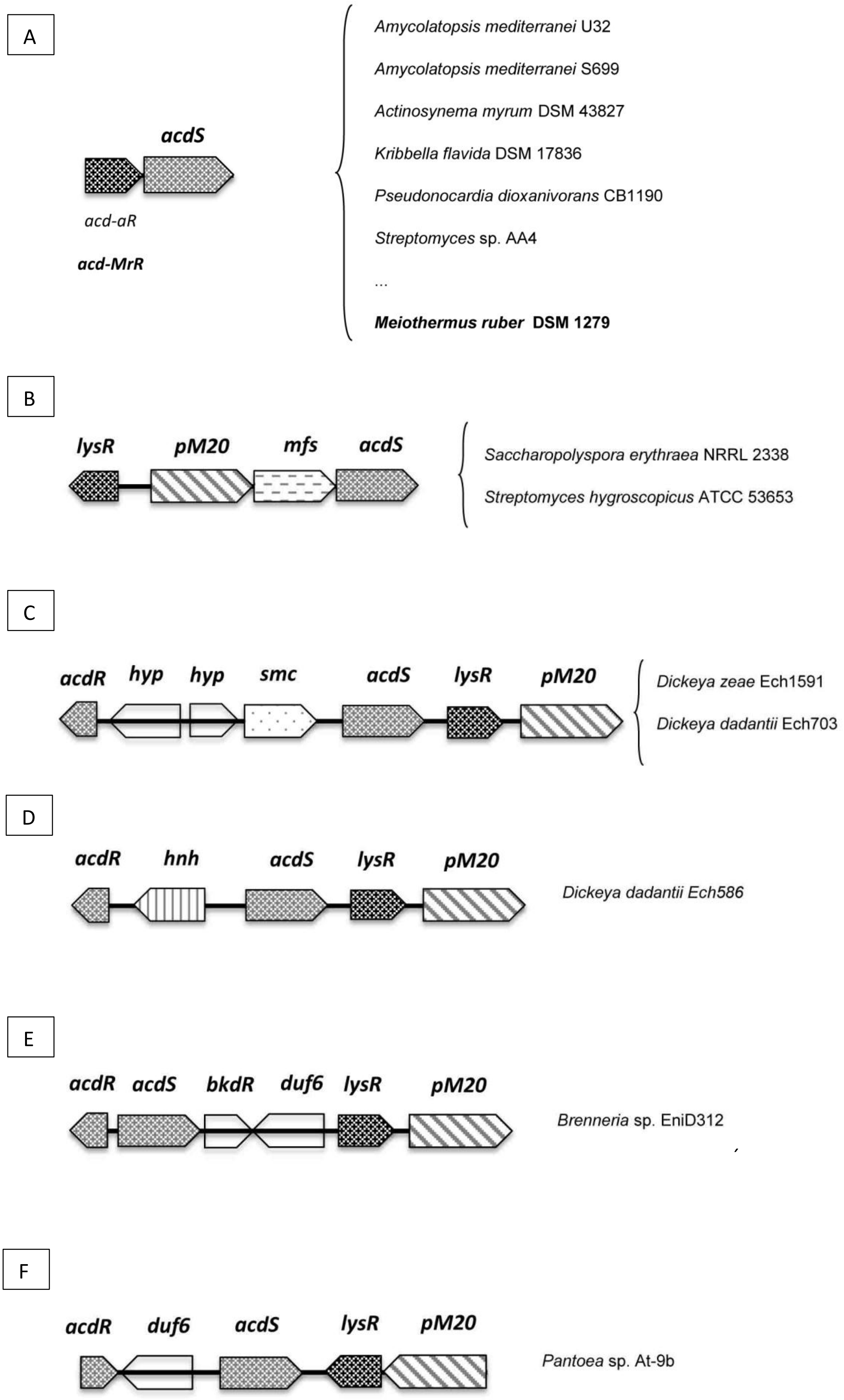

A
acdS
acd-aR
acd-MrR
Amycolatopsis mediterranei U32
Amycolatopsis mediterranei S699
Actinosynema myrum DSM 43827
Kribbella flavida DSM 17836
Pseudonocardia dioxanivorans CB1190
Streptomyces sp. AA4
...
Meiothermus ruber DSM 1279
B
lysR
pM20
mfs
acdS
Saccharopolyspora erythraea NRRL 2338
Streptomyces hygroscopicus ATCC 53653
C
acdR
hyp
hyp
smc
acdS
lysR
pM20
Dickeya zeae Ech1591
Dickeya dadantii Ech703
D
acdR
hnh
acdS
lysR
pM20
Dickeya dadantii Ech586
E
acdR
acdS
bkdR
duf6
lysR
pM20
Brenneria sp. EniD312
F
acdR
duf6
acdS
lysR
pM20
Pantoea sp. At-9b

G

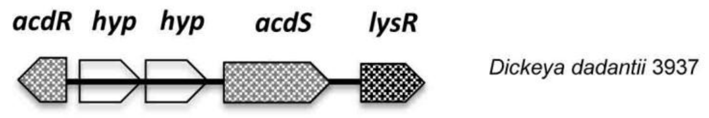
acdR hyp hyp acdS lysR
Dickeya dadantii 3937

H

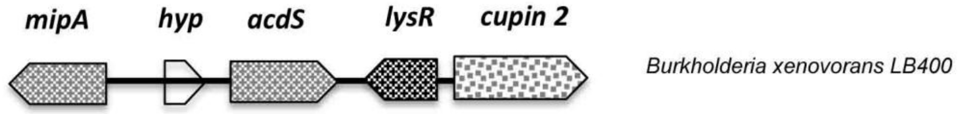
mipA hyp acdS lysR cupin 2
Burkholderia xenovorans LB400
