# Supplementary material for: New Insights into 1-Aminocyclopropane-1-Carboxylate (ACC) Deaminase Phylogeny, Evolution and Ecological Significance
Source: PLoS One. 2014 Jun 6;9(6):e99168. doi: 10.1371/journal.pone.0099168 (PMC4048297; doi:10.1371/journal.pone.0099168)
Supplement: Table S1 — Accession numbers for Actinobacteria, Deinococcus-Thermus and Firmicutes 16S rRNA, acdS and acdR genes and AcdS and AcdR protein sequences. Description of the acdS gene location, ACC deaminase (ACCD) activity, strains relative habitat and geographical origin. (DOCX) [file pone.0099168.s004.docx]

**Table S1-** Accession numbers for Actinobacteria**,** Deinococcus-Thermus and Firmicutes 16S rRNA, *acdS* and *acdR* genes and AcdS and AcdR protein sequences. Description of the *acdS* gene location, ACC deaminase (ACCD) activity, strains relative habitat and geographical origin.

| Strain | 16S rRNA | *acdS* | AcdS | *acdR*(*) | AcdR(*) | *acdS* location | ACCD  activity | Isolation/Habitat | Origin |
| --- | --- | --- | --- | --- | --- | --- | --- | --- | --- |
| *Actinoalloteichus spitiensis* RMV-1378 | AY426714.2 | AGVX02000403.1 | CT | n.a | n.a | n.a | n.a | Desert soil | India |
| *Actinoplanes missouriensis* 431 | AJ277572.1 | NC_017093.1 | YP_005461921.1 | NC_017093.1 | YP_005461920.1 | n.a | n.a | Barnyard soil | USA |
| *Actinopolyspora halophila* DSM 43834 | X54287.1 | AQUI01000002.1 | CT | n.a | n.a | n.a | n.a | Contaminant of a culture containing 25% NaCl | n.a |
| *Actinosynnema mirum* DSM 43827 | CP001630.1 | CP001630.1 | YP_003099210.1 | CP001630.1 | YP_003099209.1 | C | n.a | Grass from Raritan River | USA |
| *Agreia* sp. PHSC20C1 | AAOB01000003.1 | AAOB01000003.1 | EAR25507.1 | AAOB01000003.1 | EAR25508.1 | n.a | n.a | Marine | Antarctic |
| *Amycolatopsis azurea* DSM 43854 | AJ400709.1 | NZ_ANMG01000058.1 | ZP_21908980.1 | NZ_ANMG01000058.1 | ZP_21908979.1 | n.a | n.a | Soil | Japan |
| *Amycolatopsis decaplanina* DSM 44594 | AJ508237.1 | NZ_AOHO01000014.1 | ZP_22939816.1 | NZ_AOHO01000014.1 | ZP_22939815.1 | n.a | n.a | Soil | India |
| *Amycolatopsis mediterranei S699* | CP002896.1 | CP002896.1 | AEK44688.1 | CP002896.1 | AEK44687.1 | C | n.a | Soil sample from a pine arboretum | France |
| *Amycolatopsis mediterranei* U32 | CP002000.1 | CP002000.1 | YP_003768201.1 | CP002000.1 | YP_003768200.1 | C | n.a | Soil | n.a |
| *Amycolatopsis methanolica* 239 | AJ249135.1 | AQUL01000001.1 | CT | n.a | n.a | n.a | n.a | Soil | Papua New Guinea |
| *Amycolatopsis orientalis* HCCB10007 | n.a | CP003410.1 | AGM07658.1 | CP003410.1 | AGM07657.1 | C | n.a | n.a | n.a |
| *Amycolatopsis* sp. ATCC 39116 | AM263202.1 | NZ_JH414689.1 | ZP_10050982.1 | NZ_JH414689.1 | ZP_10050981.1 | n.a | n.a | Soil | USA |
| *Arsenicicoccus bolidensis* DSM 15745 | AJ558133.2 | AUFG01000015.1 | CT | n.a | n.a | n.a | n.a | Lake sediment containing mine waste | Sweden |
| *Arthrobacter crystallopoietes* BAB-32 | ANPE02000028.1 | NZ_ANPE02000106.1 | ZP_24038253.1 | NZ_ANPE02000106.1 | ZP_24038254.1 | n.a | n.a | Soil | India |
| *Arthrobacter sp.* 131MFCol6.1 | n.a | ARGT01000006.1 | CT | n.a | n.a | n.a | n.a | n.a | n.a |
| *Austwickia chelonae* NBRC 105200 | AJ243919.1 | NZ_BAGZ01000017.1 | ZP_10952085.1 | NZ_BAGZ01000017.1 | ZP_10952086.1 | n.a | n.a | Chelonids | Australia |
| *Bacillus cereus* AcdSPB4 | JN625722 | JN625726.1 | AEQ29826.1 | n.a | n.a | n.a | n.a | *Agaricus bisporus* casing soil | China |
| *Brevibacterium casei S18* | n.a | NZ_AMSP01000011.1 | ZP_18854273.1 | NZ_AMSP01000011.1 | ZP_18854274.1 | n.a | n.a | Human healty skin | India |
| *Brevibacterium linens* BL2 | NZ_AAGP01000013.1 | NZ_AAGP01000039.1 | ZP_05915229.1 | NZ_AAGP01000039.1 | ZP_05915228.1 | n.a | n.a | n.a | USA |
| *Brevibacterium* sp. JC43 | JF824806.1 | CAHK01000051.1 | CT | n.a | n.a | n.a | n.a | Host stool sample | France |
| *Demetria terragena* DSM 11295 | Y14152.1 | AQXW01000004.1 | CT | n.a | n.a | n.a | n.a | Frozen compost soil | Germany |
| *Ilumatobacter nonamiense* YM16-303 | AB360345.1 | BAOL01000068.1 | CT | n.a | n.a | n.a | n.a | Seashore sand | n.a |
| *Kineosphaera limosa* NBRC 100340 | AB550802.1 | NZ_BAHD01000034.1 | ZP_10941583.1 | NZ_BAHD01000034.1 | ZP_10941582.1 | n.a | n.a | Activated sludge | n.a |
| *Kribbella catacumbae* DSM 19601 | AM778575.1 | AQUZ01000035.1 | CT | n.a | n.a | n.a | n.a | Tufaceous surfaces in the catacombs of St. Callistus in Rome | Italy |
| *Kribbella flavida* DSM 17836 | CP001736.1 | CP001736.1 | ADB31588.1 | CP001736.1 | ADB31589.1 | C | n.a | Soil | China |
| *Meiothermus ruber* DSM 1279 | NC_013946.1 | CP001743.1 | YP_003506726.1 | CP001743.1 | YP_003506727.1 | C | n.a | Hot spring | Russia |
| *Meiothermus ruber* H328 | AB442017.1 | BAOR01000002.1 | CT | n.a | n.a | n.a | n.a | Hot spring | Japan |
| *Meiothermus timidus* DSM 17022 | AJ871168.1 | ARDL01000011.1 | CT | n.a | n.a | n.a | n.a | Hot spring | Portugal |
| *Microbacterium laevaniformans* OR221 | NZ_AJGR01000262.1 | NZ_AJGR01000114.1 | ZP_09922379.1 | NZ_AJGR01000114.1 | ZP_09922380.1 | n.a | n.a | Subsurface sediment | USA |
| *Microlunatus phosphovorus* NM-1 | AP012204.1 | AP012204.1 | BAK33832.1 | n.p | n.p | C | n.a | EBPR activated sludge | Japan |
| *Modestobacter marinus* BC501 | FO203431.1 | NC_017955.1 | YP_006364081.1 | NC_017955.1 | YP_006364080.1 | C | n.a | White marble surface | Italy |
| *Mycobacterium abscessus* 47J26 | AGQU01000002.1 | AGQU01000002.1 | EHB99430.1 | AGQU01000002.1 | EHB99431.1 | n.a | n.a | Sputum sample from cystic fibrosis patient | UK |
| *Mycobacterium abscessus* ATCC 19977 | NC_010397.1 | NC_010397.1 | YP_001702443.1 | NC_010397.1 | YP_001702444.1 | C | n.a | Human knee | n.a |
| *Mycobacterium abscessus subsp. bolletii BD* | AY859681.1 | NZ_AHAS01000007.1 | ZP_12993124.1 | NZ_AHAS01000007.1 | ZP_12993125.1 | n.a | n.a | Sputum of a patient | n.a |
| *Mycobacterium massiliense* CCUG 48898 | AKVF01000003.1 | NZ_AHAR01000007.1 | ZP_09409230.1 | NZ_AHAR01000007.1 | ZP_09409231.1 | n.a | n.a | Sputum of a patient | France |
| *Mycobacterium massiliense* GO 06 | NC_018150.1 | NC_018150.1 | YP_006520687.1 | NC_018150.1 | YP_006520688.1 | C | n.a | Human patient | Brazil |
| *Mycobacterium smegmatis* MC2 155 | NC_008596.1 | CP000480.1 | YP_890948.1 | CP000480.1 | YP_890946.1 | C | n.a | Human smegma | n.a |
| *Mycobacterium vaccae* ATCC 25954 | NZ_JH814714.1 | NZ_JH814693.1 | ZP_11011394.1 | n.p | n.p | n.a | n.a | n.a | n.a |
| *Nakamurella multipartita* DSM 44233 | NC_013235.1 | CP001737.1 | YP_003202162.1 | n.p | n.p | C | n.a | Activated sludge | Japan |
| *Nocardioidaceae bacterium* Broad-1 | n.a | ADVI01000084.1 | EGD41421.1 | ADVI01000084.1 | EGD41420.1 | n.a | n.a | Contaminant of *Coccidioides* genomes | n.a |
| *Propionicicella superfundia* DSM 22317 | n.a | AUIA01000003.1 | CT | n.a | n.a | n.a | n.a | Contaminated groundwater | USA |
| *Pseudonocardia dioxanivorans* CB1190 | CP002593.1 | CP002593.1 | AEA23305.1 | CP002593.1 | AEA23304.1 | C | n.a | Industrial sludge contaminated with 1,4-dioxan | n.a |
| *Rhodococcus opacus* M213 | AF095715.1 | NZ_AJYC02000133.1 | ZP_14483496.1 | NZ_AJYC02000133.1 | ZP_14483498.1 | n.a | n.a | Fuel-oil contaminated soil | USA |
| *Rhodococcus* sp. R04 | n.a | AFAQ01000515.1 | **CT** | n.a | n.a | n.a | n.a | Oil-contaminated soil | China |
| *Saccharopolyspora erythraea* NRRL 2338 | NC_009142.1 | AM420293.1 | YP_001104984.1 | n.p | n.p | C | n.a | Soil | Philippines |
| *Saccharothrix espanaensis* DSM 44229 | AF114807.1 | NC_019673.1 | YP_007035865.1 | NC_019673.1 | YP_007035864.1 | C | n.a | Soil | Spain |
| *Saxeibacter lacteus* DSM 19367 | n.a | AUFT01000005.1 | CT | n.a | n.a | n.a | n.a | Rock | South Korea |
| *Streptomyces acidiscabies* 84-104 | n.a | NZ_AHBF01000026.1 | ZP_10450903.1 | NZ_AHBF01000026.1 | ZP_10450904.1 | n.a | n.a | Potato | n.a |
| *Streptomyces albus* J1074 | n.a | NZ_ABYC01000170.1 | ZP_04701899.1 | NZ_ABYC01000170.1 | ZP_04701900.1 | n.a | n.a | n.a | n.a |
| *Streptomyces bottropensis* ATCC 25435 | AB026217.1 | NZ_KB405067.1 | ZP_23431620.1 | NZ_KB405067.1 | ZP_23431621.1 | n.a | n.a | Soil | n.a |
| *Streptomyces cf. griseus* XylebKG-1 | GL877172.1 | GL877172.1 | EGE42521.1 | GL877172.1 | EGE42520.1 | C | n.a | Ambrosia beetle | n.a |
| *Streptomyces chartreusis* NRRL 12338 | AGDE01000038 | AGDE01000072.1 | **CT** | n.a | n.a | n.a | n.a | Soil | n.a |
| *Streptomyces coelicoflavus* ZG0656 | AHGS01000024 | NZ_AHGS01001413.1 | ZP_13043759.1 | NZ_AHGS01001413.1 | ZP_13043760.1 | n.a | n.a | n.a | n.a |
| *Streptomyces davawensis* JCM 4913 | HE971709 | NC_020504.1 | YP_007525103.1 | NC_020504.1 | YP_007525102.1 | C | n.a | Soil | Phillipines |
| *Streptomyces ghanaensis* ATCC 14672 | AB184662.1 | NZ_ABYA01000438.1 | ZP_04688956.1 | NZ_ABYA01000438.1 | ZP_04688955.1 | n.a | n.a | Soil | Ghana |
| *Streptomyces griseus subsp. griseus* NBRC 13350 | AP009493.1 | AP009493.1 | BAG19783.1 | AP009493.1 | BAG19782.1 | C | n.a | Soil | n.a |
| *Streptomyces hygroscopicus* ATCC 53653 | NR_044201.1 | NZ_GG657754.1 | ZP_07299431.1 | n.p | n.p | n.a | n.a | Soil | India |
| *Streptomyces ipomoeae* 91-03 | NZ_AEJC01000674.1 | NZ_AEJC01000114.1 | ZP_19187453.1 | NZ_AEJC01000114.1 | ZP_19187449.1 | n.a | n.a | *Ipomoea batatas* | USA |
| *Streptomyces prunicolor* NBRC 13075 | AB184294.1 | BARF01000064.1 | CT | n.a | n.a | n.a | n.a | Soil | n.a |
| *Streptomyces scabiei* 87.22 | FN554889.1 | FN554889.1 | YP_003492562.1 | FN554889.1 | YP_003492561.1 | C | n.a | Soil | n.a |
| *Streptomyces* sp. AA4 | n.a | NZ_GG657746.1 | ZP_07280393.1 | NZ_GG657746.1 | ZP_07280392.1 | n.a | n.a | Soil | n.a |
| *Streptomyces* sp. PP-C42 | n.a | AEWS01000972.1 | **CT** | n.a | n.a | n.a | n.a | Marine | Baltic Sea |
| *Streptomyces* sp. R1-NS-10 | AB808756.1 | BARG01000021.1 | CT | n.a | n.a | n.a | n.a | n.a | n.a |
| *Streptomyces* sp. S4 | n.a | CADY01000046.1 | ZP_09179036.1 | CADY01000046.1 | ZP_09179037.1 | n.a | n.a | Leafcutter ant | n.a |
| *Streptomyces* sp. SS | AY507122.1 | AKXV01000001.1 | CT | n.a | n.a | n.a | n.a | Soil | China |
| *Streptomyces* sp. TOR3209 | n.a | AGNH01000445.1 | CT | n.a | n.a | n.a | n.a | Tomato rhizosphere | China |
| *Streptomyces* sp. W007 | JN180126.1 | NZ_AGSW01000117.1 | ZP_09402987.1 | NZ_AGSW01000117.1 | ZP_09402986.1 | n.a | n.a | Marine sediment | China |
| *Streptomyces sviceus* ATCC 29083 | AB184559.2 | CM000951.1 | EDY55280.1 | CM000951.1 | EDY55279.1 | C | n.a | Soil | n.a |
| *Streptomyces turgidiscabies* Car8 | NZ_AEJB01000333.1 | NZ_AEJB01000611.1 | ZP_20885367.1 | NZ_AEJB01000611.1 | ZP_20885369.1 | n.a | n.a | *Daucus carota* subsp. sativus | Japan |
| *Streptomyces venezuelae* ATCC 10712 | n.a | FR845719.1 | CCA54823.1 | FR845719.1 | CCA54824.1 | C | n.a | Soil | Venezuela |
| *Streptomyces violaceusniger* Tu 4113 | NZ_AEDI01000202.1 | NZ_AEDI01000002.1 | EFN21291.1 | n.p | n.p | n.a | n.a | Soil | n.a |
| *Streptomyces viridochromogenes* DSM 40736 | n.a | NZ_GG657757.1 | ZP_07302930.1 | NZ_GG657757.1 | ZP_07302931.1 | n.a | n.a | Soil | Cameroon |
| *Streptomyces viridochromogenes* Tue57 | n.a | NZ_AMLP01000211.1 | ZP_21112817.1 | NZ_AMLP01000211.1 | ZP_21112816.1 | n.a | n.a | n.a | n.a |
| *Streptomyces viridosporus* T7A | n.a | AJFD01000082.1 | CT | n.a | n.a | n.a | n.a | Soil | USA |
| *Tetrasphaera elongata* Lp2 | NR_024735.1 | CAIZ01000139.1 | CCH70862.1 | CAIZ01000139.1 | CCH70861.1 | n.a | n.a | Activated sludge | Japan |

**n.a**- not available, unknown; **n.p**- not present; **C**- Chromosome; **CT**-conceptual translation

* at least three types of putative ACC deaminase regulators may be found in different Actinobacteria and *Meiothermus* therefore, a new nomenclature for these genes is proposed in this work. However, the reference in the table presents only *acdR* and AcdR to simplify the visualization.
