# Supplementary material for: New Insights into 1-Aminocyclopropane-1-Carboxylate (ACC) Deaminase Phylogeny, Evolution and Ecological Significance
Source: PLoS One. 2014 Jun 6;9(6):e99168. doi: 10.1371/journal.pone.0099168 (PMC4048297; doi:10.1371/journal.pone.0099168)
Supplement: Table S2 — Accession numbers for α-Proteobacteria 16S rRNA, acdS and acdR genes and AcdS and AcdR proteins sequences. Description of the acdS gene location, ACC deaminase (ACCD) activity, strains relative habitat and origin. (DOCX) [file pone.0099168.s005.docx]

**Table S2**- Accession numbers for α-Proteobacteria 16S rRNA, *acdS* and *acdR* genes and AcdS and AcdR proteins sequences. Description of the *acdS* gene location, ACC deaminase (ACCD) activity, strains relative habitat and origin.

| Strain | 16S rRNA | *acdS* | AcdS | *acdR* | AcdR | *acdS* location | ACCD  activity | Isolation/Habitat | Origin |
| --- | --- | --- | --- | --- | --- | --- | --- | --- | --- |
| *Acidiphilium multivorum* AIU301 | AP012035.1 | AP012035.1 | YP_004284712.1 | AP012035.1 | YP_004284713.1 | C | n.a | Pyritic acid mine drainage | Japan |
| *Agrobacterium tumefaciens* D3 | HM143942.1 | AF315580.1 | AAK28496.1 | AF315580.1 | AAK28495.1 | P | Y/FL | Soil | Germany |
| *Agrobacterium vitis* S4 | NC_011989.1 | CP000634.1 | ACM38904.1 | CP000634.1 | ACM38903.1 | C2 | n.a | Soil | Hungary |
| *Amorphus coralli* DSM 19760 | DQ097300 | ARFZ01000005.1 | CT | n.a | n.a | n.a | n.a | *Fungia granulosa* | Israel |
| *Azorhizobium caulinodans* ORS 571 | AP009384.1 | AP009384.1 | BAF86265.1 | AP009384.1 | BAF86264.1 | C | n.a | Stem nodule | Senegal |
| *Azospirillum lipoferum* 4B | FQ311868.1 | DQ125242.2 | ABE66282.2 | DQ125242.2 | ACS92701.1 | P | Y/FL | Soil | France |
| *Azospirillum* sp. B510 | AP010946.1 | AP010948.1 | BAI75080.1 | AP010948.1 | BAI75081.1 | P | n.a | Soil | Japan |
| *Bradyrhizobium canariense* WSM4349 | NZ_KB890498.1 | NZ_KB890498.1 | CT | n.a | n.a | n.a | n.a | *Syrmatium glabrum* | USA |
| *Bradyrhizobium elkanii* 587 | AJJK01001957 | AJJK01000707.1 | CT | n.a | n.a | n.a | n.a | *Glycine max* | Brazil |
| *Bradyrhizobium elkanii* CCBAU 43297 | AJPW01000118 | AJPW01000023.1 | CT | n.a | n.a | n.a | n.a | *Glycine max* | China |
| *Bradyrhizobium japonicum* CCBAU 15354 | AJPX01000844 | AJPX01000049.1 | CT | n.a | n.a | n.a | n.a | *Glycine max* | China |
| *Bradyrhizobium japonicum* USDA 6 | AP012206 | NC_017249.1 | YP_005605144.1 | NC_017249.1 | YP_005605143.1 | C | n.a | *Glycine max* | Japan |
| *Bradyrhizobium japonicum* USDA110 | NC_004463.1 | NC_004463.1 | BAC45506.1 | NC_004463.1 | BAC45505.1 | C | Y/FL/BN | *Glycine max* | USA |
| *Bradyrhizobium liaoningense* CCBAU 05525 | AJQC01000887 | AJQC01000259.1 | CT | n.a | n.a | n.a | n.a | *Glycine max* | China |
| *Bradyrhizobium liaoningense* CCBAU 83689 | AJQD01000586 | AJQD01000027.1 | CT | n.a | n.a | n.a | n.a | *Glycine max* | China |
| *Bradyrhizobium oligotrophicum* S58 | JQ619230 | NC_020453.1 | YP_007513426.1 | NC_020453.1 | YP_007513425.1 | C | n.a | Paddy field soil | Japan |
| *Bradyrhizobium* sp. Btai1 | NC_009485.1 | NC_009485.1 | ABQ36190.1 | NC_009485.1 | ABQ36191.1 | C | n.a | Stem nodule | USA |
| *Bradyrhizobium* sp. CCGE-LA001 | NZ_AMCQ01000411.1 | NZ_AMCQ01000287.1 | ZP_16040407.1 | NZ_AMCQ01000287.1 | ZP_16040408.1 | n.a | n.a | *Phaseolus microcarpus* root nodules | Mexico |
| *Bradyrhizobium* sp*.* ORS 278 | NC_009445.1 | NC_009445.1 | CAL77571.1 | NC_009445.1 | CAL77570.1 | C | n.a | Stem nodule | Senegal |
| *Bradyrhizobium* sp. ORS 285 | CAFH01000247 | NZ_CAFH01000233.1 | ZP_09476143.1 | NZ_CAFH01000233.1 | ZP_09476144.1 | n.a | n.a | Stem nodule | n.a |
| *Bradyrhizobium* sp*.* ORS 375 | CAFI01000413 | NZ_CAFI01000189.1 | ZP_09421036.1 | NZ_CAFI01000189.1 | ZP_09421035.1 | n.a | n.a | *Aeschynomene indica*  stem nodule | n.a |
| *Bradyrhizobium* sp. S23321 | AP012279 | NC_017082.1 | YP_005447899.1 | NC_017082.1 | YP_005447900.1 | C | n.a | Paddy field soil | Japan |
| *Bradyrhizobium* sp*.* STM 3843 | CAFK01000252 | NZ_CAFK01000254.1 | ZP_09437058.1 | NZ_CAFK01000254.1 | ZP_09437057.1 | n.a | n.a | n.a | n.a |
| *Bradyrhizobium* sp. WSM1253 | AHMB01000041 | NZ_JH600073.1 | ZP_10084551.1 | NZ_JH600073.1 | ZP_10084552.1 | n.a | n.a | Root nodule | n.a |
| *Bradyrhizobium* sp. WSM471 | AHLW01000002 | NZ_CM001442.1 | ZP_09645255.1 | NZ_CM001442.1 | ZP_09645256.1 | n.a | n.a | Root nodule | n.a |
| *Bradyrhizobium* sp. YR681 | AKIY01000183 | NZ_AKIY01000270.1 | ZP_10583679.1 | NZ_AKIY01000270.1 | ZP_10583680.1 | n.a | n.a | *Populus deltoides* root | USA |
| *Bradyrhizobium yuanmingense* CCBAU 05623 | AJQJ01000234 | AJQJ01000325.1 | CT | n.a | n.a | n.a | n.a | *Glycine max* | China |
| *Chelatococcus* sp. GW1 | ALIQ01000242 | ALIQ01000170.1 | CT | n.a | n.a | n.a | n.a | Wastewater of a textile dye works | n.a |
| *Fodinicurvata sediminis* DSM 21159 | FJ357426 | ATVH01000016.1 | CT | n.a | n.a | n.a | n.a | Sediment | China |
| *Fulvimarina pelagi* HTCC2506 | AY178860.1 | AATP01000002.1 | EAU41874.1 | n.p | n.p | C | n.a | Marine | Sargasso Sea |
| *Gluconacetobacter xylinus* NBRC 3288 | AP012159.1 | AP012159.1 | BAK82498.1 | AP012159.1 | BAK82490.1 | C | n.a | Vinegar | n.a |
| *Gluconobacter frateurii* NBRC 101659 | AB678443.1 | NZ_BADZ01000001.1 | ZP_11374548.1 | NZ_BADZ01000001.1 | ZP_11374547.1 | n.a | n.a | Flower of *Monordica charantia* | Thailand |
| *Gluconobacter oxydans* H24 | NC_019396.1 | NC_019396.1 | YP_006983155.1 | NC_019396.1 | YP_006983156.1 | n.a | n.a | n.a | n.a |
| *Gluconobacter thailandicus* NBRC 3255 | AB178396.1 | NZ_BAON01000011.1 | ZP_23120210.1 | NZ_BAON01000011.1 | ZP_23120209.1 | n.a | n.a | Strawberry | Japan |
| *Labrenzia aggregata* IAM 12614 | NZ_AAUW01000023.1 | AAUW01000003.1 | ZP_01546258.1 | AAUW01000003.1 | ZP_01546257.1 | C | n.a | Sediment | Baltic Sea |
| *Mesorhizobium alhagi* CCNWXJ12-2 | NZ_AHAM01000052.1 | NZ_AHAM01000292.1 | ZP_09297037.1 | NZ_AHAM01000292.1 | ZP_09297038.1 | n.a | n.a | *Alhagi sparsifolia* | China |
| *Mesorhizobium amorphae* CCNWGS0123 | NZ_AGSN01000002.1 | NZ_AGSN01000010.1 | ZP_09085370.1 | n.p | n.p | n.a | n.a | Soil/root nodule | China |
| *Mesorhizobium ciceri* bv. *biserrulae* WSM1271 | NC_014923.1 | NC_014923.1 | ADV14828.1 | n.p | n.p | C/SI | n.a | Soil/root nodule | Italy |
| *Mesorhizobium loti* MAFF303099 | NC_002678.2 | NC_002678.2 | BAB52295.1 | n.p | n.p | C/SI | Y/BN | Soil/root nodule | Japan |
| *Mesorhizobium loti* R7a | n.a | AL672114.1 | CAD31305.1 | n.p | n.p | C/SI | n.a | Soil/root nodule | New Zealand |
| *Mesorhizobium mettallidurans* STM 4661 | n.a | CAAF010000045.1 | CCV12789.1 | n.p | n.p | n.a | n.a | *Anthyllis vulneraria* | France |
| *Mesorhizobium opportunistum* WSM2075 | ACZA00000000.1 | ACZA00000000.1 | EEW34025.1 | n.p | n.p | C/SI | n.a | Soil/root nodule | Australia |
| *Methylobacterium mesophilicum* SR1.6/6 | n.a | NZ_ANPA01000016.1 | ZP_23995079.1 | NZ_ANPA01000016.1 | ZP_23995080.1 | n.a | n.a | *Citrus sinensis* | Brazil |
| *Methylobacterium nodulans* ORS 2060 | CP001349.1 | CP001349.1 | ACL60323.1 | CP001349.1 | ACL60322.1 | C | n.a | Soil | Senegal |
| *Methylobacterium radiotolerans* JCM 2831 | CP001001.1 | CP001001.1 | ACB23516.1 | CP001001.1 | ACB23515.1 | C | n.a | Soil | Japan |
| *Methylobacterium* sp. 4-46 | CP000943.1 | CP000943.1 | ACA14842.1 | CP000943.1 | ACA14841.1 | C | n.a | *Lotononis bainesii* | n.a |
| *Methylobacterium* sp. 77 | n.a | ARCS01000002.1 | CT | n.a | n.a | n.a | n.a | n.a | USA |
| *Methylobacterium* sp. B34 | n.a | BADE01001099.1 | CT | n.a | n.a | n.a | n.a | n.a | n.a |
| *Methylobacterium* sp. GXF4 | n.a | NZ_AKFK01000048.1 | ZP_10354465.1 | NZ_AKFK01000048.1 | ZP_10354464.1 | n.a | n.a | *Vitis vinifera* | USA |
| *Nitratireductor indicus* C115 | AMSI01000044 | NZ_AMSI01000004.1 | ZP_11155157.1 | NZ_AMSI01000004.1 | ZP_11155156.1 | n.a | n.a | Deep seawater | Indian Ocean |
| *Pannonibacter phragmitetus* DSM 14782 | n.a | ARNQ01000046.1 | CT | n.a | n.a | n.a | n.a | Surface of decomposing rhizomes of reed | Hungary |
| *Pelagibaca bermudensis* HTCC2601 | NZ_AATQ01000003.1 | AATQ01000052.1 | EAU44226.1 | AATQ01000052.1 | EAU44227.1 | n.a | n.a | Marine | Sargasso Sea |
| *Phyllobacterium brassicacearum* STM 196 | AY785319.1 | EF452620.1 | ABO31418.1 | EF452621.1 | ABO31419.1 | n.a | Y/FL | Soil | France |
| *Rhizobium gallicum* PB2 | EF525207.1 | EF525234.1 | ABP88062.1 | EF525261.1 | ABP88045.1 | n.a | Y/FL | Soil | Canada |
| *Rhizobium grahamii* CCGE 502 | AEYE01000061 | AEYE01000046.1 | CT | n.a | n.a | n.a | n.a | *Dalea leporina* | Mexico |
| *Rhizobium leguminosarum* bv*. trifolii* SRDI565 | n.a | AQUD01000006.1 | CT | n.a | n.a | n.a | n.a | *Trifolium subterraneum* | Australia |
| *Rhizobium leguminosarum* bv. *trifolii* WU95 | n.a | NZ_JH660657.1 | ZP_18307677.1 | NZ_JH660657.1 (pseudogene) | n.a | n.a | n.a | n.a | n.a |
| *Rhizobium leguminosarum* bv. *viciae* 128C53K | n.a | AF421376.1 | AAL16088.1 | AY172673.1 | AAO17689.1 | n.a | Y/FL | Soil | n.a |
| *Rhizobium leguminosarum* bv. *viciae* 3841 | NC_008380.1 | NC_008381.1 | YP_770380.1 | n.p | n.p | P | n.a | Soil | U.K |
| *Rhizobium leguminosarum* bv. *viciae* Vc2 | n.a | ARDP01000039.1 | CT | n.a | n.a | n.a | n.a | *Viccia cracca* | England |
| *Rhizobium leguminosarum* PB171 | EF525228.1 | EF525246.1 | ABP88074.1 | EF525273.1 | ABP88057.1 | n.a | Y/FL | Soil | Canada |
| *Rhizobium leguminosarum* PB223 | EF525233.1 | EF525250.1 | ABP88078.1 | EF525277.1 | ABP88061.1 | n.a | Y/FL | Soil | Canada |
| *Rhizobium leguminosarum* PB62 | EF525210.1 | EF525237 | ABP88065.1 | EF525264 | ABP88048.1 | n.a | Y/FL | Soil | Canada |
| *Rhizobium mesoamericanum* STM3625 | CANI01000030 | CANI01000090.1 | CT | n.a | n.a | n.a | n.a | *Mimosa pudica* | French Guiana |
| *Rhizobium radiobacter* K84 | NC_011985.1 | CP000629.1 | ACM30009.1 | CP000629.1 | ACM30008.1 | C2 | n.a | Soil | n.a |
| *Rhizobium* sp. AP16 | AJVM01000087 | NZ_AJVM01000009.1 | ZP_10535789.1 | NZ_AJVM01000009.1 | ZP_10535790.1 | n.a | n.a | *Populus deltoides* root | USA |
| *Rhizobium* sp. PDO1-076 | AHZC01000107 | NZ_AHZC01000377.1 | ZP_13492557.1 | NZ_AHZC01000377.1 | ZP_13492558.1 | n.a | n.a | *Populus deltoides* root | USA |
| *Rhizobium* sp. Pop5 | AMCP01001078 | NZ_AMCP01000592.1 | ZP_16036138.1 | NZ_AMCP01000592.1 | ZP_16036139.1 | n.a | n.a | *Phaseolus vulgaris* root nodule | Mexico |
| *Rhizobium* sp. PRF 81 | n.a | AQHN01000086.1 | CT | n.a | n.a | n.a | n.a | *Phaseolus vulgaris* | Brazil |
| *Rhizobium tropici* CIAT 899 | HQ850704 | NC_020061.1 | YP_007336138.1 | n.p | n.p | P | n.a | *Phaseolus vulgaris* root nodule | Colombia |
| *Roseibium* sp. Trich SKD4 | NZ_GL476310.1 | NZ_GL476315.1 | ZP_07659928.1 | NZ_GL476315.1 | ZP_07659927.1 | C | n.a | Seawater, Trichodesmium colonies | North Atlantic Ocean |
| *Sagittula stellata* E-37 | NZ_AAYA00000000 | AAYA01000005.1 | EBA08640.1 | AAYA01000005.1 | EBA08641.1 | C | n.a | Coastal seawater | USA |
| *Salipiger mucosus* DSM 16094 | n.a | ARRM01000068.1 | CT | n.a | n.a | n.a | n.a | Saline soil bordering a saltern | Spain |
| *Sinorhizobium fredii* GR64 | AMCX01000136 | AMCX01000095.1 | CT | n.a | n.a | C | n.a | *Phaseolus vulgaris* | Spain |
| *Sinorhizobium medicae* WSM419 | NC_009636.1 | NC_009622 | YP_001314953.1 | NC_009622 | YP_001314954.1 | P | n.a | Soil | Italy |
| *Sinorhizobium meliloti* 4H41 | n.a | AQWP01000042.1 | CT | n.a | n.a | n.a | n.a | n.a | n.a |
| *Sinorhizobium meliloti* AK83 | NZ_AEDH01000067.1 | NZ_AEDH01000067.1 | ZP_07601419.1 | NZ_AEDH01000067.1 | ZP_07601418.1 | C3 | n.a | Soil | Kazakhstan |
| *Sinorhizobium meliloti* BL225C | AEDG01000070.1 | AEDG01000070.1 | ZP_07593541.1 | AEDG01000070.1 | ZP_07593542.1 | P | n.a | Soil | Italy |
| *Sinorhizobium meliloti* CCNWSX0020 | AGVV01000154 | NZ_AGVV01000056.1 | ZP_12976532.1 | NZ_AGVV01000056.1 | ZP_12976531.1 | n.a | n.a | *Medicago lupulina* | China |
| *Sinorhizobium meliloti* KYA40 | EU603723.1 | EU603722.1 | ACC78287.1 | n.a | n.a | n.a | n.a | Soil | Iran |
| *Sinorhizobium meliloti* KYA71 | EU603721.1 | EU003994.1 | ABS19884.1 | n.a | n.a | n.a | n.a | Soil | Iran |
| *Sinorhizobium meliloti* SM11 | CP001830.1 | DQ145546.1 | ABA56046.1 | DQ145546.1 | ABA56047.1 | P | Y/FL | Soil | Germany |
| *Sinorhizobium* sp. BL3 | AY943949.1 | EU183545.1 | ABW39374.1 | EU183545.1 | ABW39373.1 | n.a | Y/FL | Soil | Thailand |
| *Starkeya novella* DSM 506 | NR_025859.1 | CP002026.1 | ADH87862.1 | NC_014217.1 | ADH87863.1 | C | n.a | Soil | n.a |
| *Thalassospira xiamenensis* DSM 17429 | n.a | NZ_AMRQ01000015.1 | ZP_11118220.1 | NZ_AMRQ01000015.1 | ZP_11118219.1 | n.a | n.a | Surface water of a waste oil pool | China |
| *Tistrella mobilis* KA081020-065 | CP003236 | CP003239.1 | AFK57065.1 | CP003239.1 | AFK57066.1 | P | n.a | Marine | Red Sea |

**n.a**- not available; **n.p**- not present; **C**- Chromosome; **C2**- 2^nd^ chromosome; **C/SI**- Chromosome/symbiotic island;  **P**- plasmid; **Y/FL**- Yes/free living conditions; **Y/BN**- Yes/ bacteroid state (nitrogen fixing); **n.a-** unknown. **CT**- conceptual translation.
