# Supplementary material for: New Insights into 1-Aminocyclopropane-1-Carboxylate (ACC) Deaminase Phylogeny, Evolution and Ecological Significance
Source: PLoS One. 2014 Jun 6;9(6):e99168. doi: 10.1371/journal.pone.0099168 (PMC4048297; doi:10.1371/journal.pone.0099168)
Supplement: Table S3 — Accession numbers for β-Proteobacteria 16S rRNA, acdS and acdR genes and AcdS and AcdR proteins sequences and description of the acdS gene location, ACC deaminase (ACCD) activity, strains relative habitat and origin. (DOCX) [file pone.0099168.s006.docx]

**Table S3-** Accession numbers for β-Proteobacteria 16S rRNA, *acdS* and *acdR* genes and AcdS and AcdR proteins sequences and description of the *acdS* gene location, ACC deaminase (ACCD) activity, strains relative habitat and origin.

| Strain | 16S rRNA | *acdS* | AcdS | *acdR* | AcdR | *acdS* location | ACCD  activity | Isolation/Habitat | Origin |
| --- | --- | --- | --- | --- | --- | --- | --- | --- | --- |
| *Achromobacter arsenitoxydans*  SY8 | NZ_AGUF01000004.1 | NZ_AGUF01000042.1 | ZP_09299986.1 | NZ_AGUF01000042.1 | ZP_09299985.1 | n.a | n.a | Soil contaminated with arsenic | China |
| *Achromobacter piechaudii* HLE | ALJE01000119 | NZ_ALJE01000008.1 | ZP_15930486.1 | NZ_ALJE01000008.1 | ZP_15930487.1 | n.a | n.a | Soil | USA |
| *Achromobacter xylosoxidans* A8 | CP002287.1 | CP002287.1 | YP_003977696.1 | CP002287.1 | YP_003977697.1 | C | n.a | Soil contaminated with polychlorinated biphenyls | Czech Republic |
| *Achromobacter xylosoxidans* C54 | ACRC01000072.1 | ACRC01000687.1 | EFV82431.1 | ACRC01000687.1 | EFV82432.1 | n.a | n.a | Cystic fibrosis patient | n.a |
| *Acidovorax avenae* subsp. *avenae* ATCC 19860 | CP002521.1 | CP002521.1 | YP_004236837.1 | CP002521.1 | YP_004236836.1 | C | n.a | Maize leaf | USA |
| *Acidovorax citrulli* AAC00-1 | CP000512.1 | CP000512.1 | YP_972804.1 | CP000512.1 | YP_972803.1 | C | n.a | Watermellon | n.a |
| *Acidovorax ebreus* TPSY | CP001392.1 | CP001392.1 | YP_002554583.1 | CP001392.1 | YP_002554582.1 | C | n.a | Groundwater, soil | USA |
| *Acidovorax radicis* N35 | NZ_AFBG01000030.1 | NZ_AFBG01000023.1 | ZP_08950254.1 | NZ_AFBG01000023.1 | ZP_08950253.1 | n.a | n.a | Wheat roots | Germany |
| *Acidovorax* sp. CF316 | AKJX01000236 | AKJX01000025.1 | EJE54366.1 | AKJX01000025.1 | EJE54367.1 | n.a | n.a | *Populus deltoides* | USA |
| *Acidovorax* sp. JS42 | CP000539.1 | CP000539.1 | YP_988060.1 | CP000539.1 | YP_988059.1 | C | n.a | Nitrobenzene-contaminated sediment | USA |
| *Acidovorax* sp. KKS102 | CP003872 | NC_018708.1 | YP_006856666.1 | NC_018708.1 | YP_006856665.1 | C | n.a | Soil | Japan |
| *Bordetella* sp. FB-8 | JN885794 | ARNH01000003.1 | CT | n.a | n.a | n.a | n.a | Sediment | Germany |
| *Burkholderia ambifaria* AMMD | CP000441.1 | CP000441.1 | YP_777038.1 | CP000441.1 | YP_777039.1 | C2 | n.a | Pea rhizosphere | USA |
| *Burkholderia ambifaria* MC40-6 | NC_010552.1 | NC_010552.1 | YP_001809994.1 | NC_010552.1 | YP_001809995.1 | C2 | Y/FL | Soil associated with maize roots | USA |
| *Burkholderia caledonica*  LMG 19076 | AF215704.1 | EU886299.1 | ACH81521.1 | n.a | n.a | n.a | Y/FL | Rhizosphere soil | Scotland |
| *Burkholderia caryophylli* LMG 2155 | AB021423.1 | EU886300.1 | ACH81522.1 | n.a | n.a | n.a | Y/FL | *Dianthus caryophyllus* | USA |
| *Burkholderia cenocepacia* HI2424 | CP000459.1 | CP000459.1 | ABK10113.1 | CP000459.1 | ABK10114.1 | C2 | n.a | Onion field | USA |
| *Burkholderia cenocepacia* J2315 | AM747721.1 | AM747721.1 | YP_002233009.1 | AM747721.1 | YP_002233010.1 | C2 | Y/FL | Cystic fibrosis patient | Scotland |
| *Burkholderia cenocepacia* MC0-3 | CP000959.1 | CP000959.1 | YP_001777783.1 | CP000959.1 | YP_001777782.1 | C2 | n.a | Soil associated with maize roots | USA |
| *Burkholderia cenocepacia* PC184 | n.a | NZ_CH482379.1 | ZP_04944067.1 | n.a | n.a | n.a | n.a | Cystic fibrosis patient | USA |
| *Burkholderia cepacia* ATCC 25416 | n.a | EU886301.1 | ACH81523.1 | n.a | n.a | n.a | Y/FL | Onion | USA |
| *Burkholderia dolosa* AUO158 | n.a | NZ_CH482381.1 | ZP_04948196.1 | NZ_CH482381.1 | ZP_04948197.1 | n.a | n.a | Cystic fibrosis patient | USA |
| *Burkholderia gladioli* BSR3 | n.a | CP002600.1 | AEA63740.1 | CP002600.1 | AEA63739.1 | C2 | n.a | Diseased rice sheath | South Korea |
| *Burkholderia glumae* BGR1 | n.a | CP001504.1 | YP_002908968.1 | CP001504.1 | YP_002908969.1 | C2 | n.a | Diseased rice panicle | Korea |
| *Burkholderia graminis* C4D1M | U96939.1 | EU886302.1 | ACH81524.1 | NZ_ABLD01000001.1 | ZP_02881625.1 | n.a | Y/FL | Senescent maize roots | France |
| *Burkholderia mallei* ATCC 23344 | NC_006349.2 | NC_006349.2 | YP_105635.1 | NC_006349.2 | YP_105634.1 | C2 | n.a | Glanders-melioidosis patient | Burma |
| *Burkholderia mallei* NCTC 10229 | NC_008835.1 | NC_008835.1 | YP_001024043.1 | NC_008835.1 | YP_001024042.1 | C2 | n.a | n.a | Hungary |
| *Burkholderia mallei* PRL-20 | NZ_AAZP01000007.1 | AAZP01000009.1 | ZP_02265430.1 | AAZP01000009.1 | ZP_02265429.1 | n.a | n.a | Horse blood | Pakistan |
| *Burkholderia multivorans* ATCC 17616 | AP009386.1 | AP009386.1 | YP_001585177.1 | AP009386.1 | YP_001585176.1 | C2 | n.a | Soil | n.a |
| *Burkholderia multivorans* CGD1 | NZ_ACFB01000007.1 | ACFB01000002.1 | ZP_03583630.1 | ACFB01000002.1 | ZP_03583631.1 | n.a | n.a | Chronic granulomatous disease patient | USA |
| *Burkholderia oklahomensis* C6786 | NZ_ABBG01000575.1 | NZ_ABBG01000392.1 | ZP_02365950.1 | NZ_ABBG01000392.1 | ZP_02365951.1 | n.a | n.a | Human leg wound | USA |
| *Burkholderia phenoliruptrix* BR3459a | CP003863 | NC_018672.1 | YP_006792732.1 | NC_018672.1 | YP_006792731.1 | C2 | n.a | *Mimosa flocculosa* | Brazil |
| *Burkholderia phenoliruptrix* BR3459a | - | NC_018696.1 | YP_006836544.1 | n.p | n.p | P | n.a | - | - |
| *Burkholderia phenoliruptrix* LMG 22037 | AY435213.1 | EU886303.1 | ACH81525.1 | n.a | n.a | n.a | Y/FL | Soil | n.a |
| *Burkholderia phymatum* STM815 | CP001044.1 | CP001044.1 | YP_001861528.1 | CP001044.1 | YP_001861529.1 | C2 | n.a | Root nodule | French Guiana |
| *Burkholderia phymatum* STM815 | - | CP001046.1 | YP_001863743.1 | n.p | n.p | P | n.a | - | - |
| *Burkholderia phytofirmans* PsJN | CP001053.1 | CP001053.1 | YP_001889125.1 | CP001053.1 | YP_001889124.1 | C2 | Y/FL | Onion roots | n.a |
| *Burkholderia pseudomallei* 1106b | AAMB02000036 | NZ_CM000775.1 | ZP_04811587.1 | NZ_CM000775.1 | ZP_04811063.1 | C2 | n.a | Human liver abcess | Thailand |
| *Burkholderia pseudomallei* 1710b | NC_007435.1 | YP_335503.1 | NC_007435.1 | YP_335504.1 | YP_335504.1 | C2 | n.a | Human Blood | Thailand |
| *Burkholderia pseudomallei* 668 | CP000571.1 | CP000571.1 | YP_001062875.1 | CP000571.1 | YP_001062876.1 | C2 | n.a | Melioidosis patient | Australia |
| *Burkholderia pyrrocinia* CH-67 | ALWI01000067 | ALWI01000021.1 | CT | n.a | n.a | n.a | n.a | Forest soil | South Korea |
| *Burkholderia silvatlantica* AB48 | AF164043.2 | EU886305.1 | ACH81527.1 | n.a | n.a | n.a | Y/FL | Pineapple roots | Brazil |
| *Burkholderia silvatlantica* PPCR-2 | AY965243.1 | EU886304.1 | ACH81526.1 | n.a | n.a | n.a | Y/FL | Sugarcane roots | Brazil |
| *Burkholderia silvatlantica* SRMrh-20 | AY965240.1 | EU886306.1 | ACH81528.1 | n.a | n.a | n.a | Y/FL | Maize roots | Brazil |
| *Burkholderia* sp. 383 | CP000152.1 | CP000152.1 | YP_373615.1 | CP000152.1 | YP_373614.1 | C2 | n.a | Forest soil | Trinidad |
| *Burkholderia* sp. BT03 | AKKD01000264 | AKKD01000037.1 | EJL64220.1 | AKKD01000037.1 | EJL64219.1 | n.a | n.a | *Populus deltoides* | USA |
| *Burkholderia* sp. CCGE1001 | CP002520.1 | CP002520.1 | YP_004230185.1 | CP002520.1 | YP_004230184.1 | C2 | n.a | n.a | n.a |
| *Burkholderia* sp. CCGE1002 | NC_014118.1 | NC_014118.1 | YP_003607498.1 | NC_014118.1 | YP_003607497.1 | C2 | n.a | Soil, root nodule | Mexico |
| *Burkholderia* sp. CCGE1002 | - | CP002016.1 | ADG20824.1 | n.p | n.p | P | n.a | - | - |
| *Burkholderia* sp*.* CCGE1003 | NC_014539.1 | CP002218.1 | ADN59699.1 | CP002218.1 | ADN59698.1 | C2 | n.a | n.a | n.a |
| *Burkholderia* sp. Ch1-1 | NZ_ADNR01000109.1 | NZ_ADNR01000001.1 | ZP_06838775.1 | NZ_ADNR01000001.1 | ZP_06838776.1 | n.a | n.a | PAH contaminated soil | USA |
| *Burkholderia* sp. H160 | NZ_ABYL01000300.1 | NZ_ABYL01000102.1 | ZP_03269041.1 | NZ_ABYL01000102.1 | ZP_03269042.1 | n.a | n.a | n.a | n.a |
| *Burkholderia* sp. SJ98 | AJHK02000001 | NZ_AJHK02000011.2 | ZP_11399945.1 | NZ_AJHK02000011.2 | ZP_11399944.1 | n.a | n.a | Soil | India |
| *Burkholderia* sp. TJI49 | n.a | AEXE01000286.1 | EGD05602.1 | AEXE01000286.1 | EGD05601.1 | n.a | n.a | Bark of mango tree | Pakistan |
| *Burkholderia* sp*.* YI23 | CP003089.1 | CP003089.1 | AET93258.1 | CP003089.1 | AET93257.1 | C3 | n.a | Soil | South Korea |
| *Burkholderia terrae* BS001 | AKAU01000146 | NZ_AKAU01000015.1 | ZP_10247444.1 | NZ_AKAU01000015.1 | ZP_10247445.1 | n.a | n.a | Soil underneath mushroom foot | Netherlands |
| *Burkholderia terricola* LMG 20594 | AY040362.1 | EU886307.1 | ACH81529.1 | n.a | n.a | n.a | Y/FL | Soil | n.a |
| *Burkholderia thailandensis* E264 | CP000085.1 | CP000085.1 | YP_439298.1 | CP000085.1 | YP_439297.1 | C2 | n.a | Environmental isolate (Soil) | Thailand |
| *Burkholderia thailandensis* MSMB43 | NZ_ABBM01000203.1 | NZ_ABBM01000778.1 | ZP_02466645.1 | NZ_ABBM01000778.1 | ZP_02466646.1 | n.a | n.a | Borehole | Australia |
| *Burkholderia thailandensis* TXDOH | NZ_ABBD01000779.1 | NZ_ABBD01000124.1 | ZP_02370572.1 | NZ_ABBD01000124.1 | ZP_02370571.1 | n.a | n.a | Human blood | USA |
| *Burkholderia ubonensis* Bu | ABBE01000728.1 | NZ_ABBE01001097.1 | ZP_02383018.1 | NZ_ABBE01001097.1 | ZP_02383017.1 | n.a | n.a | Rhizosphere sample from a mine site | Australia |
| *Burkholderia unamae*CAC-98 | n.a | EU886308.1 | ACH81530.1 | n.a | n.a | n.a | Y/FL | Cofee plant rhizosphere | Mexico |
| *Burkholderia unamae* MTI-641 | AY221956.1 | EU886320.1 | ACH81542.1 | EU886320.1 | ACH81543.1 | n.a | Y/FL | Maize rhizosphere | Mexico |
| *Burkholderia vietnamiensis* G4 | CP000615.1 | CP000615.1 | YP_001116376.1 | CP000615.1 | YP_001116377.1 | C2 | n.a | Industrial waste treatment facility | USA |
| *Burkholderia vietnamiensis* LMG 6999 | n.a | EU886310.1 | ACH81532.1 | n.a | n.a | n.a | Y/FL | Human neck abcess | n.a |
| *Burkholderia vietnamiensis* TVV75 | U96928.1 | EU886309.1 | ACH81531.1 | n.a | n.a | n.a | Y/FL | Acid sulphate soil | Vietnam |
| *Burkholderia xenovorans* CAC-124 | n.a | EU886312.1 | ACH81534.1 | n.a | n.a | n.a | Y/FL | Cofee plant rhizosphere | Mexico |
| *Burkholderia xenovorans* CCUG 28445 | n.a | EU886313.1 | ACH81535.1 | n.a | n.a | n.a | Y/FL | Human blood | Sweden |
| *Burkholderia xenovorans* LB400 | NC_007952.1 | NC_007952.1 | YP_554094.1 | NC_007952.1 | YP_554434.1 | C2 | Y/FL | PCB-containing landfill | USA |
| *Collimonas fungivorans* Ter331 | NC_015856.1 | NC_015856.1 | YP_004752723.1 | NC_015856.1 | YP_004752724.1 | C | n.a | Soil | Netherlands |
| *Cupriavidus basilensis* OR16 | AHJE01000064 | AHJE01000053.1 | EHP41147.1 | AHJE01000053.1 | EHP41148.1 | n.a | n.a | Pristine soil | Hungary |
| *Cupriavidus necator* N-1 | NC_015726.1 | CP002878.1 | AEI80287.1 | CP002878.1 | AEI80288.1 | C2 | n.a | Soil | USA |
| *Cupriavidus* sp. UYPR2.512 | JF683703 | ARBE01000348.1 | CT | n.a | n.a | n.a | n.a | *Parapiptadenia rigida* root nodule | Uruguay |
| *Cupriavidus* sp. UYPR2.512 | - | ARBE01000173.1 | CT | n.a | n.a | n.a | n.a | - | - |
| *Curvibacter lanceolatus* ATCC 14669 | AB021390.1 | ARLO01000035.1 | CT | n.a | n.a | n.a | n.a | Distilled water | Canada |
| *Herbaspirillum frisingense* GSF30 | AEEC01001472 | NZ_AEEC01000097.1 | ZP_11552248.1 | NZ_AEEC01000097.1 | ZP_11552250.1 | n.a | Y/FL | *Miscanthus* | Germany |
| *Herbaspirillum huttiense subsp. putei* IAM 15032 | AB109890 | ANJR01000014.1 | CT | n.a | n.a | n.a | n.a | Well water | Japan |
| *Herbaspirillum lusitanum* P6-12 | AJHH01000137 | AJHH01000633.1 | CT | n.a | n.a | n.a | n.a | *Phaseolus vulgaris* root nodule | Brazil |
| *Herbaspirillum seropedicae* SmR1 | CP002039.1 | CP002039.1 | ADJ64675.1 | CP002039.1 | ADJ64674.1 | C | n.a | *Sorghum bicolor* roots | Brazil |
| *Herbaspirillum* sp. B501 | AB049133 | BADJ01001136.1 | CT | n.a | n.a | n.a | n.a | *Oryza officinalis* | Japan |
| *Herbaspirillum* sp. CF444 | AKJW01000112 | NZ_AKJW01000044.1 | ZP_10721003.1 | NZ_AKJW01000044.1 | ZP_10721002.1 | n.a | n.a | *Populus deltoides* | USA |
| *Herbaspirillum* sp. GW103 | AJVC01000004 | NZ_AJVC01000004.1 | ZP_11255386.1 | NZ_AJVC01000004.1 | ZP_11255384.1 | n.a | n.a | Rhizosphere soil | South Korea |
| *Herbaspirillum* sp. YR522 | AKJA01000016 | NZ_AKJA01000043.1 | ZP_10591596.1 | NZ_AKJA01000043.1 | ZP_10591597.1 | n.a | n.a | *Populus deltoides* | USA |
| *Methylibium petroleiphilum* PM1 | CP000555.1 | CP000555.1 | YP_001022786.1 | CP000555.1 | YP_001022785.1 | C | n.a | Biolfilter in an oil refinery | USA |
| *Polaromonas* sp. CF318 | AKIV01000055 | NZ_AKIV01000015.1 | ZP_10561707.1 | NZ_AKIV01000015.1 | ZP_10561708.1 | n.a | n.a | *Populus deltoides* root | USA |
| *Polaromonas* sp. JS666 | CP000316.1 | CP000316.1 | YP_550624.1 | CP000316.1 | YP_550625.1 | C | n.a | Contaminated groundwater | USA |
| *Ralstonia eutropha* H16 | AM260480.1 | AM260480.1 | YP_840884.1 | AM260480.1 | YP_840885.1 | C2 | n.a | Spring | Germany |
| *Ralstonia pickettii* 12D | NC_012856.1 | NC_012856.1 | YP_002981652.1 | NC_012856.1 | YP_002981651.1 | C | n.a | Copper-contaminated sediment from a lake | USA |
| *Ralstonia pickettii* 12J | CP001068.1 | CP001068.1 | YP_001899571.1 | CP001068.1 | YP_001899570.1 | C | n.a | Copper-contaminated sediment from a lake | USA |
| *Ralstonia solanacearum* CFBP2957 | FP885897.1 | FP885907.1 | YP_003747920.1 | FP885907.1 | YP_003747919.1 | P | n.a | Tomato | French West Indie |
| *Ralstonia solanacearum* CMR15 | FP885895.1 | FP885896.1 | CBJ40249.1 | FP885896.1 | CBJ40248.1 | P | n.a | Tomato | Cameroon |
| *Ralstonia solanacearum* GMI1000 | NC_003295.1 | AL646053.1 | NP_522207.1 | AL646053.1 | NP_522206.1 | P | Y/FL | Tomato | Guiana |
| *Ralstonia solanacearum* Po82 | CP002819.1 | CP002820.1 | AEG71624.1 | CP002820.1 | AEG71623.1 | P | n.a | Potato | Mexico |
| *Ralstonia solanacearum* PSI07 | NC_014311.1 | FP885891.2 | YP_003749510.1 | FP885891.2 | YP_003749509.1 | P | n.a | Tomato | Indonesia |
| *Ralstonia solanacearum* Y45 | n.a | AFWL01000379.1 | CT | n.a | n.a | P | n.a | Tobacco plant | China |
| *Ralstonia* sp. 5_2_56FAA | ACTT01000008.1 | ACTT01000035.1 | EGY64300.1 | ACTT01000035.1 | EGY64299.1 | n.a | n.a | Patient with Crohn's disease | n.a |
| *Ralstonia* sp. 5_7_47FAA | ACUF01000076.1 | NZ_ACUF01000054.1 | ZP_07677216.1 | NZ_ACUF01000054.1 | ZP_07677217.1 | n.a | n.a | Patient with Crohn's disease | n.a |
| *Ralstonia syzygii* R24 | FR854086.1 | FR854090.1 | CCA87810.1 | FR854090.1 | CCA87809.1 | n.a | n.a | Diseased clove tree | Indonesia |
| *Variovorax paradoxus* 5C2 | n.a | AY604531.2 | AAT35829.2 | n.a | n.a | n.a | Y/FL | Mining waste soil | Russia |
| *Variovorax paradoxus* EPS | CP002417.1 | CP002417.1 | YP_004158083.1 | CP002417.1 | YP_004158082.1 | C | n.a | Soil | n.a |
| *Variovorax paradoxus* S110 | CP001635.1 | CP001635.1 | YP_002946967.1 | CP001635.1 | YP_002946966.1 | C | n.a | Potato plant | USA |
| *Variovorax* sp. CF313 | AKIW01000103 | AKIW01000011.1 | EJL79371.1 | AKIW01000011.1 | EJL79370.1 | n.a | n.a | *Populus deltoides* | USA |

**n.a**- not available, unknown; **n.p**- not present; **C**- Chromosome; **C2**- 2^nd^ chromosome;  **P**- plasmid; **Y/FL**- Yes/free living conditions;
