# Supplementary material for: New Insights into 1-Aminocyclopropane-1-Carboxylate (ACC) Deaminase Phylogeny, Evolution and Ecological Significance
Source: PLoS One. 2014 Jun 6;9(6):e99168. doi: 10.1371/journal.pone.0099168 (PMC4048297; doi:10.1371/journal.pone.0099168)
Supplement: Table S4 — Accession numbers for γ-Proteobacteria 16S rRNA, acdS and acdR genes and AcdS and AcdR proteins sequences and description of the acdS gene location, ACC deaminase (ACCD) activity, strains relative habitat and origin. (DOCX) [file pone.0099168.s007.docx]

**Table S4**- Accession numbers for γ-Proteobacteria 16S rRNA, *acdS* and *acdR* genes and AcdS and AcdR proteins sequences and description of the *acdS* gene location, ACC deaminase (ACCD) activity, strains relative habitat and origin.

| Strain | 16S rRNA | *acdS* | AcdS | *acdR* | AcdR | *acdS* location | ACCD  activity | Isolation/Habitat | Origin |
| --- | --- | --- | --- | --- | --- | --- | --- | --- | --- |
| *Brenneria sp.* EniD312 | AFWW01000001 | NZ_CM001230.1 | ZP_09017848.1 | NZ_CM001230.1 | ZP_09017849.1 | C | n.a | Plant | n.a |
| *Dickeya dadantii* 3937 | CP002038.1 | CP002038.1 | YP_003881235.1 | CP002038.1 | YP_003881238.1 | C | n.a | *Saintpaulia ionantha* plants | France |
| *Dickeya dadantii* Ech586 | NC_013592.1 | NC_013592.1 | YP_003332054.1 | NC_013592.1 | YP_003332056.1 | C | n.a | n.a | n.a |
| *Dickeya dadantii* Ech703 | NC_012880.1 | NC_012880.1 | YP_002989081.1 | NC_012880.1 | YP_002989077.1 | C | n.a | n.a | n.a |
| *Dickeya dianthicola* NCPPB 3534 | n.a | AOOK01000005.1 | CT | n.a | n.a | n.a | n.a | n.a | n.a |
| *Dickeya paradisiaca* NCPPB 2511 | Z96096.1 | AONV01000036.1 | CT | n.a | n.a | n.a | n.a | n.a | n.a |
| *Dickeya solani* MK10 | n.a | AOOP01000004.1 | CT | n.a | n.a | n.a | n.a | n.a | n.a |
| *Dickeya zeae* Ech1591 | CP001655.1 | CP001655.1 | YP_003005910.1 | NC_012912.1 | YP_003005906.1 | C | n.a | n.a | n.a |
| *Halomonas boliviensis* LC1 | AGQZ01000062 | NZ_JH393257.1 | ZP_09188008.1 | NZ_JH393257.1 | ZP_09188009.1 | n.a | n.a | Soil around the hypersaline lake Laguna Colorada | Bolivia |
| *Halomonas* sp. HAL1 | EU651835.1 | AGIB01000084.1 | EHA14104.1 | AGIB01000084.1 | EHA14105.1 | C | n.a | Soil from a gold mine | China |
| *Halomonas* sp. KM-1 | HD061326 | NZ_BAEU01000110.1 | ZP_10778750.1 | NZ_BAEU01000110.1 | ZP_10778748.1 | C | n.a | n.a | Japan |
| *Halomonas stevensii* S18214 | AJTS01000020 | AJTS01000040.1 | CT | n.a | n.a | n.a | n.a | Blood from a renal care patient | USA |
| *Halomonas titanicae* BH1 | n.a | NZ_AOPO01000001.1 | ZP_21727375.1 | n.p | n.p | n.a | n.a | Rusticles of the RMS Titanic wreck | Atlantic ocean |
| *Klebsiella oxytoca* Rs-5 | n.a | FJ357241.1 | ACJ12921.1 | n.a | n.a | n.a | n.a | n.a | n.a |
| *Klebsiella pneumoniae* AcdSPB2 | JN625720 | JN625725.1 | AEQ29825.1 | n.a | n.a | n.a | Y/FL | *Agaricus bisporus* casing soil | China |
| *Pantoea* sp. At-9b | CP002433.1 | CP002436.1 | ADU72453.1 | CP002436.1 | ADU72455.1 | P | n.a | *Atta cephalotes* (leaf cutter ant)symbiont | n.a |
| *Pseudomonas avellanae* BPIC 631 | AKBS01001374 | NZ_JH951555.1 | ZP_16386983.1 | NZ_JH951555.1 | ZP_16386982.1 | n.a | n.a | *Corylus avellana* | Greece |
| *Pseudomonas brassicacearum* subsp. brassicacearum NFM421 | CP002585.1 | CP002585.1 | AEA68459.1 | CP002585.1 | AEA68458.1 | C | n.a | n.a | n.a |
| *Pseudomonas entomophila* PS-PJH | n.a | FJ882923.1 | ACQ55296.1 | n.a | n.a | n.a | Y/FL | Red pepper rhizosphere | Korea |
| *Pseudomonas fluorescens* 17 | n.a | U37103.1 | AAC44163.1 | n.a | n.a | n.a | Y/FL | Soil | South Africa |
| *Pseudomonas fluorescens* 2P24 | AY447045.1 | EF635249.1 | ABR26447.1 | EF635249.1 | ABR26446.1 | n.a | n.a | Wheat take-all decline soil | China |
| *Pseudomonas fluorescens* F113 | CP003150.1 | CP003150.1 | AEV63500.1 | CP003150.1 | AEV63499.1 | n.a | Y/FL | Sugarbeet rhizosphere | n.a |
| *Pseudomonas fluorescens* FY32 | FJ465156.2 | FJ465155.1 | ACJ69586.1 | n.a | n.a | P | Y/FL | Soil | Iran |
| *Pseudomonas fuscovaginae* UPB0736 | AIEU01000016 | NZ_JH605158.1 | ZP_10991581.1 | NZ_JH605158.1 | ZP_10991582.1 | n.a | n.a | Sheath brown rot lesion on rice | Madagascar: Antsirabe |
| *Pseudomonas psychrotolerans* L19 | NZ_AHBD01000036.1 | NZ_AHBD01000009.1 | ZP_09285984.1 | NZ_AHBD01000009.1 | ZP_09285985.1 | n.a | n.a | Copper alloy coins | n.a |
| *Pseudomonas putida* AKMP7 | GU396282.1 | HM053973.1 | ADH59751.1 | n.a | n.a | n.a | n.a | Sorghum rhizosphere | India |
| *Pseudomonas putida* AM15 | EF194770.1 | EF011160.1 | ABJ91236.1 | n.a | n.a | n.a | n.a | n.a | India |
| *Pseudomonas putida* AS1.1003 | n.a | EU700088.1 | ACD70372.1 | n.a | n.a | n.a | n.a | n.a | China |
| *Pseudomonas savastanoi* pv. savastanoi NCPPB 3335 | NZ_GG774664.1 | GG774632.1 | EFI00387.1 | GG774632.1 | EFI00388.1 | n.a | n.a | Diseased olive tree | France |
| *Pseudomonas* sp. 313 | n.a | ANBZ01000041.1 | CT | n.a | n.a | n.a | n.a | Kelp holobiont | USA |
| *Pseudomonas* sp. 6G5 | n.a | M80882.1 | AAA73153.1 | n.a | n.a | n.a | Y/FL | Soil | n.a |
| *Pseudomonas* sp. ACP | n.a | n.a | Q00740.1 | n.a | n.a | n.a | Y/FL | Soil | Japan |
| *Pseudomonas* sp. AT14 | EF194771.1 | EF011161.1 | ABJ91237.1 | n.a | n.a | n.a | n.a | n.a | India |
| *Pseudomonas* sp. CH-GRS 8 | n.a | EF581137.1 | ABQ10596.1 | n.a | n.a | n.a | n.a | n.a | India |
| *Pseudomonas* sp. GM102 | NZ_AKJB01000132.1 | NZ_AKJB01000115.1 | ZP_10599704.1 | n.p | n.p | n.a | n.a | *Populus deltoides* | USA |
| *Pseudomonas* sp. GM18 | AKJT01000077 | NZ_AKJT01000072.1 | ZP_10705137.1 | n.p | n.p | n.a | n.a | *Populus deltoides* | USA |
| *Pseudomonas* sp. GM55 | AKJJ01000082 | NZ_AKJJ01000032.1 | ZP_10643223.1 | n.p | n.p | n.a | n.a | *Populus deltoides* | USA |
| *Pseudomonas* sp. GM67 | AKJH01000024 | NZ_AKJH01000180.1 | ZP_10636320.1 | NZ_AKJH01000180.1 | ZP_10636321.1 | n.a | n.a | *Populus deltoides* | USA |
| *Pseudomonas* sp. GM79 | AKJE01000062 | NZ_AKJE01000083.1 | ZP_10616599.1 | n.p | n.p | n.a | n.a | *Populus deltoides* | USA |
| *Pseudomonas* sp. PNSL | n.a | DQ830987.1 | ABH03031.1 | n.a | n.a | n.a | n.a | n.a | Taiwann.a |
| *Pseudomonas* sp. Ps 2-3 | n.a | EU520401.1 | ACA97076.1 | n.a | n.a | n.a | n.a | n.a | India |
| *Pseudomonas* sp. Ps 7-12 | n.a | EU520398.1 | ACA97075.1 | n.a | n.a | n.a | n.a | n.a | India |
| *Pseudomonas* sp. UW4 | AY559493.1 | AY823987.1 | AAV73804.1 | AY686539.1 | AAU00683.1 | C | Y/FL | Soil | Canada |
| *Pseudomonas syringae* BRIP39023 | n.a | AMZX01000054.1 | ELQ07806.1 | AMZX01000054.1 | ELQ07805.1 | n.a | n.a | Barley | Australia |
| *Pseudomonas syringae* ICMP 18806 | n.a | ANJF01000119.1 | CT | n.a | n.a | n.a | n.a | n.a | New Zealand |
| *Pseudomonas syringae pv. aceris* M302273 | AEAO01000730 | AEAO01000438.1 | CT | n.a | n.a | n.a | n.a | Maple | n.a |
| *Pseudomonas syringae pv. actinidiae* CRAFRU8.43 | AFTG01000283 | AFTG01000155.1 | CT | n.a | n.a | n.a | n.a | *Actinidia deliciosa* | Italy |
| *Pseudomonas syringae pv. actinidiae* M302091 | AEAL01000630 | AEAL01000023.1 | CT | n.a | n.a | n.a | n.a | *Actinidia deliciosa* | Japan |
| *Pseudomonas syringae pv. aesculi* 2250 | ACXT01000186 | ACXT01000276.1 | CT | n.a | n.a | n.a | n.a | *Aesculus hippocastanum* | Scotland |
| *Pseudomonas syringae* *pv. aesculi* NCPPB3681 | NZ_ACXS01000064.1 | NZ_ACXS01000074.1 | ZP_06457192.1 | NZ_ACXS01000074.1 | ZP_06457191.1 | C | n.a | *Aesculus indica* | India |
| *Pseudomonas syringae pv. aptata* DSM 50252 | AEAN01001255.1 | AEAN01001087.1 | EGH80018.1 | AEAN01001087.1 | EGH80019.1 | n.a | n.a | Sugarbeet | n.a |
| *Pseudomonas syringae pv. avellanae* ISPaVe037 | AKCK01000070 | NZ_JH951881.1 | ZP_17808950.1 | NZ_JH951881.1 | ZP_17808951.1 | n.a | n.a | *Corylus avellana* | Italy |
| *Pseudomonas syringae pv. glycinea* B076 | AEGG01000013.1 | AEGG01000041.1 | EFW79594.1 | AEGG01000041.1 | EFW79593.1 | C | n.a | Diseased soybean leaflet | USA |
| *Pseudomonas syringae pv. glycinea* race 4 | AEGH01000005.1 | AEGH01000057.1 | EFW85440.1 | AEGH01000057.1 | EFW85441.1 | n.a | n.a | n.a | n.a |
| *Pseudomonas syringae pv. japonica* M301072 | AEAH01001400 | NZ_GL384842.1 | ZP_16686047.1 | NZ_GL384842.1 | ZP_16686046.1 | n.a | n.a | n.a | n.a |
| *Pseudomonas syringae pv. lachrymans* M302278 | AEAM01000561.1 | AEAM01000159.1 | EGH95996.1 | AEAM01000159.1 | EGH95995.1 | n.a | n.a | n.a | n.a |
| *Pseudomonas syringae* *pv. maculicola* ES4326 | AEAK01000607.1 | AEAK01000297.1 | EGH60475.1 | AEAK01000297.1 | EGH60476.1 | n.a | n.a | n.a | n.a |
| *Pseudomonas syringae pv. mori* 301020 | AEAG01001117.1 | AEAG01000549.1 | EGH22588.1 | AEAG01000549.1 | EGH22587.1 | n.a | n.a | n.a | n.a |
| *Pseudomonas syringae pv. morsprunorum* M302280 | AEAE01000512 | AEAE01000519.1 | CT | n.a | n.a | n.a | n.a | n.a | n.a |
| *Pseudomonas syringae* *pv. oryzae* 1_6 | NZ_ABZR01000729.1 | NZ_ABZR01000328.1 | ZP_04588011.1 | NZ_ABZR01000328.1 | ZP_04588012.1 | n.a | n.a | n.a | n.a |
| *Pseudomonas syringae pv. panici* LMG 2367 | ALAC01000003 | ALAC01000004.1 | CT | n.a | n.a | n.a | n.a | n.a | n.a |
| *Pseudomonas syringae* *pv. phaseolicola* 1448A | CP000058.1 | CP000058.1 | AAZ35072.1 | CP000058.1 | AAZ33570.1 | C | n.a | *P. vulgaris* | Ethiopia |
| *Pseudomonas syringae* *pv. syringae* 642 | NZ_ADGB01000251.1 | NZ_ADGB01000177.1 | ZP_07265622.1 | NZ_ADGB01000177.1 | ZP_07265623.1 | n.a | n.a | Plant | USA |
| *Pseudomonas syringae* *pv. syringae* B728a | CP000075.1 | CP000075.1 | AAY36848.1 | CP000075.1 | AAY36849.1 | C | n.a | Snap bean leaflet | USA |
| *Pseudomonas syringae* pv. syringae FF5 | NZ_ACXZ01002235.1 | NZ_ACXZ01004270.1 | ZP_06500448.1 | NZ_ACXZ01004270.1 | ZP_06500449.1 | n.a | n.a | Bradford pear | n.a |
| *Pseudomonas syringae pv. tabaci* 6605 | AJXI01000282 | AJXI01000213.1 | CT | n.a | n.a | n.a | n.a | n.a | n.a |
| *Pseudomonas syringae pv. theae* NCPPB 2598 | AGNN01000645 | AGNN01000508.1 | CT | n.a | n.a | n.a | n.a | n.a | n.a |
| *Pseudomonas syringae* *pv. tomato* DC3000 | AE016853.1 | AE016853.1 | AAO57144.1 | AE016853.1 | AAO57143.1 | C | n.a | Tomato | UK |
| *Pseudomonas syringae* *pv. tomato* NCPPB 1108 | n.a | NZ_ADGA01000205.1 | ZP_07260367.1 | NZ_ADGA01000205.1 | ZP_07260368.1 | n.a | n.a | Tomato | UK |
| *Pseudomonas viridiflava* UASWS0038 | AMQP01000017 | NZ_AMQP01000152.1 | ZP_11290060.1 | NZ_AMQP01000152.1 | ZP_11290059.1 | n.a | n.a | Infected *Rhododendron* sp. leaf | Switzerland |
| *Serratia rubidea* AcdSPB1 | JN625719 | JN625724.1 | AEQ29824.1 | n.a | n.a | n.a | Y/FL | *Agaricus bisporus* casting soil | China |
| *Serratia* sp. M24T3 | HQ538811 | NZ_AJHJ01000044.1 | ZP_09972421.1 | NZ_AJHJ01000044.1 | ZP_09972422.1 | n.a | n.a | *Bursaphelenchus xylophilus* | Portugal |
| *Vibrio gazogenes* ATCC 43941 | n.a | ASAJ01000012.1 | CT | n.a | n.a | n.a | n.a | Saltwater marsh | USA |

**n.a**- not available, unknown; **n.p**- not present; **C**- Chromosome;  **P**- plasmid; **Y/FL**- Yes/free living conditions; **CT**- conceptual translation
