# Supplementary material for: New Insights into 1-Aminocyclopropane-1-Carboxylate (ACC) Deaminase Phylogeny, Evolution and Ecological Significance
Source: PLoS One. 2014 Jun 6;9(6):e99168. doi: 10.1371/journal.pone.0099168 (PMC4048297; doi:10.1371/journal.pone.0099168)
Supplement: Table S5 — Accession numbers for Eukaryotes AcdS complete sequences and description of ACC deaminase (ACCD) activity, strains relative habitat and geographical origin. (DOCX) [file pone.0099168.s008.docx]

**Table S5-** Accession numbers for Eukaryotes AcdS complete sequences and description of ACC deaminase (ACCD) activity, strains relative habitat and geographical origin.

| Strain | AcdS | ACCD  activity | Isolation/Habitat | Origin |
| --- | --- | --- | --- | --- |
| *Arthrobotrys oligospora* ATCC 24927 | EGX50717.1 | n.a | Dung of livestock | n.a |
| *Arthroderma benhamiae* CBS 112371 | XP_003015331.1 | n.a | Patient with inflammatory epidermophytosis | Switzerland |
| *Arthroderma gypseum* CBS 118893 | XP_003172451.1 | n.a | Soil | n.a |
| *Arthroderma otae* CBS 113480 | XP_002843926.1 | n.a | Human patient | Germany |
| *Aspergillus flavus* NRRL3357 | XP_002378560.1 | n.a | Peanut cotyledons | USA |
| *Aspergillus fumigatus* A1163 | EDP53767.1 | n.a | Clinical isolate | n.a |
| *Aspergillus fumigatus* Af293 | XP_749239.1 | n.a | Human patient | UK |
| *Aspergillus kawachii* IFO 4308 | GAA91871.1 | n.a | n.a | Japan |
| *Aspergillus oryzae* RIB40 | XP_001823215.1 | n.a | Cereal | Japan |
| *Aureobasidium pullulans* AY4 | AMCU01000114.1 (CT) | n.a | Human skin sample | Malaysia |
| *Beauveria bassiana* ARSEF 2860 | EJP66687.1 | n.a | Insect | n.a |
| *Chaetomium thermophilum* var. thermophilum DSM 1495 | EGS18697.1 | n.a | Decaying wheat straw | UK |
| *Clavispora lusitaniae* ATCC 42720 | XP_002616765.1 | n.a | Human blood | USA |
| *Coccidioides immitis* RS | XP_001248460.1 | n.a | Human patient | USA |
| *Coccidioides posadasii* C735 delta SOWgp | XP_003070780.1 | n.a | Human patient | n.a |
| *Coccidioides posadasii* Silveira | EFW22190.1 | n.a | Human patient | USA |
| *Colletotrichum higginsianum* IMI 349063 | CCF37171.1 | n.a | *Brassica rapa* | Trinidad |
| *Colletotrichum orbiculare* MAFF 240422 | ENH86277.1 | n.a | *Cucumis sativus* | Japan |
| *Cordyceps militaris* CM01 | EGX91007.1 | n.a | n.a | n.a |
| *Cryptococcus neoformans* *var. neoformans* B-3501ª | XP_777022.1 | n.a | n.a | Laboratory strain |
| *Cryptococcus neoformans* *var. neoformans* JEC21 | XP_568760.1 | n.a | n.a | Laboratory strain |
| *Cyberlindnera saturnus* | Q7M523.1 | Y/FL | Soil | Japan |
| *Cyberlindnera jadinii* NBRC 0988 | BAEL01000113.1 (CT) | n.a | n.a | n.a |
| *Dothistroma septosporum* NZE10 | EME39015.1 | n.a | *Pinus radiata* | New Zealand |
| *Drosophila eugracilis* | AFPQ01002869.1 (CT) | n.a | n.a | n.a |
| *Eutypa lata* UCREL1 | EMR61327.1 | n.a | n.a | n.a |
| *Exophiala dermatitidis* NIH/UT8656 | EHY53954.1 | n.a | n.a | n.a |
| *Fomitopsis pinicola* FP-58527 | AEHC01000092.1  (CT) | n.a | Conifer tree | n.a |
| *Fusarium oxysporum f. sp. cubense* race 4 | EMT60768.1 | n.a | n.a | n.a |
| *Fusarium pseudograminearum* CS3096 | EKJ74886.1 | n.a | n.a | Australia |
| *Gaeumannomyces graminis var. tritici* R3-111a-1 | EJT70645.1 | n.a | Roots | n.a |
| *Gibberella zeae* PH-1 | XP_385209.1 | n.a | Wheat kernels | USA |
| *Glomerella graminicola* M1.001 | EFQ25139.1 | n.a | Maize | USA |
| *Grosmannia clavigera* kw1407 | EFX01604.1 | n.a | *Dendroctonus ponderosae* symbiont | n.a |
| *Guignardia citricarpa* CGMCC3.14348 | AOTE01003224.1 (CT) | n.a | Leaf | China |
| *Howardula aeoronymphium* | CT * | n.a | *Drosophila* parasite | n.a |
| *Hyaloperonospora arabidopsidis* Emoy2 | ABWE01000578.1 (CT) | n.a | *Arabidopsis thaliana* | n.a |
| *Macrophomina phaseolina* MS6 | EKG09749.1 | n.a | *Corchorus olitorius* | Bangladesh |
| *Magnaporthe oryzae* 70-15 | XP_001522461.1 | n.a | Rice | n.a |
| *Marssonina brunnea f. sp. 'multigermtubi'* MB_m1 | EKD13448.1 | n.a | Poplar tree | China |
| *Metarhizium acridum* CQMa 102 | EFY85645.1 | n.a | n.a | n.a |
| *Myceliophthora thermophila* ATCC 42464 | AEO56095.1 | n.a | Soil | n.a |
| *Mycosphaerella graminicola* IPO323 | EGP82604.1 | n.a | Wheat | Netherlands |
| *Mycosphaerella populorum* SO2202 | EMF16135.1 | n.a | Poplar tree | n.a |
| *Nectria haematococca* mpVI 77-13-4 | XP_003045841.1 | n.a | n.a | n.a |
| *Neosartorya fischeri* NRRL 181 | XP_001265664.1 | n.a | Canned apples | n.a |
| *Neurospora crassa* OR74A | XP_959200.2 | n.a | n.a | n.a |
| *Neurospora tetrasperma* FGSC 2508 | EGO61449.1 | n.a | n.a | USA |
| *Penicillium chrysogenum* Wisconsin 54-1255 | XP_002566393.1 | n.a | n.a | n.a |
| *Penicillium citrinum* | BAA92150.1 | Y/FL | n.a | n.a |
| *Penicillium digitatum* Pd1 | EKV05343.1 | n.a | Grapefruit | Spain |
| *Penicillium marneffei* ATCC 18224 | XP_002152267.1 | n.a | Bamboo rat, *Rhizomys sinensis* | Vietnam |
| *Phytophthora infestans* T30-4 | XP_002999006.1 | n.a | Potato | Netehrlands |
| *Phytophthora kernoviae* 00844/4 | AOFK01000115.1 (CT) | n.a | *Rhododendron ponticum* | UK |
| *Phytophthora lateralis* | AOFH01000927.1  (CT) | n.a | *Chamaecyparis lawsoniana* | USA |
| *Phytophthora parasitica* P1976 | ANJA01000726.1  (CT) | n.a | n.a | n.a |
| *Phytophthora ramorum* Pr102 | AAQX01001189.1 (CT) | n.a | *Quercus agrifolia* | USA |
| *Phytophthora sojae* P6497 | EGZ05423.1 | n.a | Soybean | USA |
| *Pseudocercospora fijiensis* CIRAD86 | EME86929.1 | n.a | Banana | n.a |
| *Pseudoperonospora cubensis* MSU-1 | AHJF01000354.1 (CT) | n.a | *Cucumis sativus* | USA |
| *Punctularia strigosozonata* HHB-11173 SS5 | EIN03908.1 | n.a | n.a | n.a |
| *Schizophyllum commune* H4-8 | XP_003028947.1 | n.a | n.a | n.a |
| *Schizosaccharomyces pombe* 972h- | NP_595003.1 | n.a | n.a | Laboratory strain |
| *Sordaria macrospora* k-hell | XP_003347375.1 | n.a | n.a | n.a |
| *Talaromyces stipitatus* ATCC 10500 | XP_002487215.1 | n.a | Rotting wood | USA |
| *Thielavia terrestris* NRRL 8126 | AEO62504.1 | n.a | n.a | n.a |
| *Trichoderma asperellum* T203 | ACX94231.1 | Y/FL | n.a | n.a |
| *Trichoderma atroviride* IMI 206040 | EHK47723.1 | n.a | Soil | Sweden |
| *Trichoderma reesei* QM6a | EGR46173.1 | n.a | Tent canvas | Solomon Islands |
| *Trichoderma virens* Gv29-8 | EHK17293.1 | n.a | Agricultural soil | USA |
| *Trichophyton equinum* CBS 127.97 | EGE01138.1 | n.a | Human patient | Finland |
| *Trichophyton rubrum* CBS 118892 | XP_003237222.1 | n.a | Human patient | Germany |
| *Trichophyton tonsurans* CBS 112818 | EGD96016.1 | n.a | Human patient | Canada |
| *Trichophyton verrucosum* HKI 0517 | XP_003021717.1 | n.a | Human patient | n.a |
| *Verticillium dahliae* VdLs.17 | EGY20763.1 | n.a | Lettuce | California |

**n.a-** not available, unknown**; (CT)**- conceptual translation.

* Conceptual translation from the sequence obtained in *Howardula* *aeoronymphium* genome database. (<http://nematodes.org/downloads/959nematodegenomes/blast/db/Howardula_aoronymphium_clc_1.fna>) (contig_97899).
