# Supplementary material for: New Insights into 1-Aminocyclopropane-1-Carboxylate (ACC) Deaminase Phylogeny, Evolution and Ecological Significance
Source: PLoS One. 2014 Jun 6;9(6):e99168. doi: 10.1371/journal.pone.0099168 (PMC4048297; doi:10.1371/journal.pone.0099168)
Supplement: Table S6 — Acession numbers for the sequences used in Figure 5 . (DOCX) [file pone.0099168.s009.docx]

**Table 6**- Acession numbers for the sequences used in Figure 5.

| Organism | P | Acession |
| --- | --- | --- |
| *Acaryochloris* sp. CCMEE 5410 | Bacteria; Cyanobacteria | WP_010475890.1 |
| *Alkaliphilus metalliredigens* | Bacteria; Firmicutes | YP_001318478.1 |
| *Amphimedon queenslandica* | Eukaryota; Metazoa | XP_003383707.1 |
| *Arabidopsis lyrata subsp. lyrata* | Eukaryota; Viridiplantae | XP_002894112.1 |
| *Arabidopsis thaliana* | Eukaryota; Viridiplantae | AAF79717.1 |
| *Bacillus bataviensis* | Bacteria; Firmicutes | WP_007087484.1 |
| *Bacillus cereus* | Bacteria; Firmicutes | WP_016115337.1 |
| *Bacillus megaterium* DSM 319 | Bacteria; Firmicutes | YP_003599085.1 |
| *Bacillus thuringiensis* | Bacteria; Firmicutes | WP_001046607.1 |
| *Bordetella bronchiseptica* MO149 | Bacteria; Proteobacteria (Beta) | YP_006902444.1 |
| *Branchiostoma floridae* | Eukaryota; Metazoa | XP_002591482.1 |
| *Calditrix abyssi* | Bacteria; Caldithrix | WP_006929754.1 |
| *Capitella teleta* | Eukaryota; Metazoa | ELT93489.1 |
| *Caulobacter crescentus* CB15 | Bacteria; Proteobacteria alpha | NP_420839.1 |
| *Chlamydomonas reinhardtii* | Eukaryota; Viridiplantae | XP_001700834.1 |
| *Chlorella variabilis* | Eukaryota; Viridiplantae | EFN58625.1 |
| *Cicer arietinum* | Eukaryota; Viridiplantae | XP_004503246.1 |
| *Ciona intestinalis* | Eukaryota; Metazoa | XP_002121189.1 |
| *Clostridium symbiosum* | Bacteria; Firmicutes | WP_003503279.1 |
| *Coccomyxa subellipsoidea* C-169 | Eukaryota; Viridiplantae | EIE24755.1 |
| *Coprobacillus* sp. 8_2_54BFAA | Bacteria; Firmicutes | WP_008792520.1 |
| *Cronobacter sakazakii* | Bacteria; Proteobacteria (gamma) | WP_007901773.1 |
| *Deinococcus deserti* VCD115 | Bacteria; Deinococcus-Thermus | YP_002787455.1 |
| *Dinoroseobacter shibae* DFL 12 | Bacteria; Proteobacteria (alpha) | YP_001532782.1 |
| *Emiliania huxleyi* | Eukaryota; Haptophyceae | EOD31262.1 |
| *Erwinia tasmaniensis* Et1/99 | Bacteria; Proteobacteria (gamma) | YP_001907367.1 |
| *Escherichia coli* K-12 | Bacteria; Proteobacteria (gamma) | YP_490176.1 |
| *Fragaria vesca subsp. vesca* | Eukaryota; Viridiplantae | XP_004299485.1 |
| *Fusobacterium ulcerans* | Bacteria; Fusobacteria | WP_005978024.1 |
| *Glycine max* | Eukaryota; Viridiplantae | XP_003525175.1 |
| *Haliangium ochraceum* | Bacteria; Proteobacteria delta | YP_003269644.1 |
| *Johnsonella ignava* | Bacteria; Firmicutes | WP_005540296.1 |
| *Lactobacillus parafarraginis* | Bacteria; Firmicutes | WP_008211299.1 |
| *Luminiphilus syltensis* | Bacteria; Proteobacteria (gamma) | WP_009019997.1 |
| *Methylobacterium populi* BJ001 | Bacteria; Proteobacteria (alpha) | YP_001925011.1 |
| *Nematostella vectensis* | Eukaryota; Metazoa | XP_001637312.1 |
| *Oceanibaculum indicum* | Bacteria; Proteobacteria (alpha) | WP_008945246.1 |
| *Oikopleura dioica* | Eukaryota; Metazoa | CBY35070.1 |
| *Pectobacterium carotovorum* | Bacteria; Proteobacteria (gamma) | WP_010285227.1 |
| *Phytophthora infestans* T30-4 | Eukaryota; Stramenopiles | XP_002906856.1 |
| *Phytophthora sojae* | Eukaryota; Stramenopiles | EGZ28718.1 |
| *Prunus persica* | Eukaryota; Viridiplantae | EMJ10357.1 |
| *Pseudoalteromonas atlantica* T6c | Bacteria; Proteobacteria (gamma) | YP_662233.1 |
| *Pseudomonas fluorescens* F113 | Bacteria; Proteobacteria (gamma) | YP_005205697.1 |
| *Pseudomonas putida* UW4 | Bacteria; Proteobacteria (gamma) | YP_007027206.1 |
| *Pseudomonas stutzeri* | Bacteria; Proteobacteria (gamma) | WP_003291494.1 |
| *Psychrobacter arcticus* 273-4 | Bacteria; Proteobacteria (gamma) | YP_264886.1 |
| *Pyrococcus horikoshii* OT3 | Archaea; Euryarchaeota | NP_142071.2 |
| *Ramlibacter tataouinensis* TTB310 | Bacteria; Proteobacteria (Beta) | YP_004619850.1 |
| *Roseobacter* sp. SK209-2-6 | Bacteria; Proteobacteria (alpha) | EBA18139.1 |
| *Ruegeria pomeroyi* DSS-3 | Bacteria; Proteobacteria (alpha) | AAV95902.1 |
| *Salmonella typhimurium* LT2 | Bacteria; Proteobacteria (gamma) | AAL20865.1 |
| *Selaginella moellendorffii* | Eukaryota; Viridiplantae | XP_002961916.1 |
| *Simiduia agarivorans* | Bacteria; Proteobacteria (gamma) | YP_006915655.1 |
| *Solanum lycopersicum* | Eukaryota; Viridiplantae | NP_001234368.1 |
| *Staphylococcus pettenkoferi* | Bacteria; Firmicutes | WP_002470882.1 |
| *Strongylocentrotus purpuratus* | Eukaryota; Metazoa; | NP_001229618.1 |
| *Syntrophobotulus glycolicus* | Bacteria; Firmicutes | YP_004265300.1 |
| *Teredinibacter turnerae* T7901 | Bacteria; Proteobacteria (gamma) | YP_003073255.1 |
| *Thalassiosira oceanica* | Eukaryota; Stramenopiles | EJK45891.1 |
| *Thermococcus sp. AM4* | Archaea; Euryarchaeota | YP_002582067.2 |
| *Thermotoga maritima MSB8* | Bacteria; Thermotogae | NP_228040.1 |
| *Trichoplax adhaerens* | Eukaryota; Metazoa | XP_002109431.1 |
| *Triticum urartu* | Eukaryota; Viridiplantae | EMS48554.1 |
| *Vibrio splendidus* | Bacteria; Proteobacteria (gamma) | WP_004739369.1 |
| *Vibrio tubiashii* | Bacteria; Proteobacteria (gamma) | WP_004744649.1 |
| *Volvox carteri f. nagariensis* | Eukaryota; Viridiplantae | XP_002955139.1 |
| *Zea mays* | Eukaryota; Viridiplantae | NP_001130254.1 |
